# Bacterial Genes Encoding Resistance Against Antibiotics and Metals in Well-Maintained Drinking Water Distribution Systems in Finland

**DOI:** 10.3389/fmicb.2021.803094

**Published:** 2022-02-07

**Authors:** Ananda Tiwari, Vicente Gomez-Alvarez, Sallamaari Siponen, Anniina Sarekoski, Anna-Maria Hokajärvi, Ari Kauppinen, Eila Torvinen, Ilkka T. Miettinen, Tarja Pitkänen

**Affiliations:** ^1^Expert Microbiology Unit, Finnish Institute for Health and Welfare, Kuopio, Finland; ^2^Department of Food Hygiene and Environmental Health, Faculty of Veterinary Medicine, University of Helsinki, Helsinki, Finland; ^3^Office of Research and Development, U.S. Environmental Protection Agency, Cincinnati, OH, United States; ^4^Department of Environmental and Biological Sciences, University of Eastern Finland, Kuopio, Finland

**Keywords:** drinking water distribution system, drinking water treatment, disinfection, metagenomics, mercury resistant gene, antibiotic resistance genes

## Abstract

Information on the co-occurrence of antibiotic resistance genes (ARGs) and metal resistance genes (MRGs) among bacterial communities in drinking water distribution systems (DWDSs) is scarce. This study characterized ARGs and MRGs in five well-maintained DWDSs in Finland. The studied DWDSs had different raw water sources and treatment methods. Two of the waterworks employed artificially recharged groundwater (ARGW) and used no disinfection in the treatment process. The other three waterworks (two surface and one groundwater source) used UV light and chlorine during the treatment process. Ten bulk water samples (two from each DWDS) were collected, and environmental DNA was extracted and then sequenced using the Illumina HiSeq platform for high-throughput shotgun metagenome sequencing. A total of 430 ARGs were characterized among all samples with the highest diversity of ARGs identified from samples collected from non-disinfected DWDSs. Furthermore, non-disinfected DWDSs contained the highest diversity of bacterial communities. However, samples from DWDSs using disinfectants contained over double the ratio of ARG reads to 16S rRNA gene reads and most of the MRG (namely mercury and arsenic resistance genes). The total reads and types of ARGs conferring genes associated with antibiotic groups namely multidrug resistance, and bacitracin, beta-lactam, and aminoglycoside and mercury resistance genes increased in waterworks treating surface water with disinfection. The findings of this study contribute toward a comprehensive understanding of ARGs and MRGs in DWDSs. The occurrence of bacteria carrying antibiotic or metal resistance genes in drinking water causes direct exposure to people, and thus, more systematic investigation is needed to decipher the potential effect of these resistomes on human health.

## Introduction

Antibiotic-resistant bacteria (ARB) are a critically emerging burden for public health (Ferri et al., 2017; Ghosh et al., 2021). The World Health Organization has declared antimicrobial resistance (AMR) as one of the top ten global public health threats facing humanity (WHO, 2020). AMR is the ability of microbes to withstand the effect of antimicrobial medicines, which inevitably leads to complications in the treatment of infectious diseases. In addition, the outcomes of such complications, such as a higher mortality rate and prolonged treatment and hospitalization time all increase the heavy economic burden of AMR (Ashbolt et al., 2013; Amarasiri et al., 2020; WHO, 2020).

Antibiotic-Resistant Bacteria and antibiotic resistance genes (ARGs) have long been reported in drinking water distribution systems (DWDSs) (Xi et al., 2009; Sanganyado and Gwenzi, 2019; Chen et al., 2020; Ramos et al., 2020). ARGs enter the DWDS despite the multiple barriers of the drinking water treatment process (DWTP) *via* mobile genetic elements (MGE) and dead or viable cells (Xi et al., 2009; Chen et al., 2020). Furthermore, biofilm growth in DWDSs provides shelter for a wide taxonomic range of (micro)organisms to survive against disinfectants (Lehtola et al., 2007; Gomez-Alvarez et al., 2014, 2015, 2016; Inkinen et al., 2019, 2021; Chen et al., 2020). ARB communities in biofilms can easily detach from biofilms to the bulk water due to water force and shear stress (Amarasiri et al., 2020). Biofilms function as a hotspot for horizontal gene transfer (HGT) events and dissemination of ARGs from ARB to antibiotic susceptible microbes or even to opportunistic pathogens (Xi et al., 2009; Pal et al., 2015; Chen et al., 2020). HGT is the primary mechanism for spreading ARGs among bacterial communities through the process of transformation, transduction, or conjugation and is mediated by MGE (van Hoek et al., 2011; Bengtsson-Palme et al., 2014; Sultan et al., 2018).

Chlorination and chloramination are common disinfection practices at DWTP used in many countries, including Finland. Disinfection at the DWTP ensures biological stability and reduces biological diversity in the DWDS (Inkinen et al., 2019, 2021). However, disinfection with chlorine only affects chlorine-susceptible micro-organisms, and chlorine residuals in the distribution system might not be sufficient to control the activity of biofilm communities. Further, ultraviolet (UV) disinfection in DWTP is also a common practice, considered as simple, reliable, and cost-effective approach effective for some chlorine-resistant pathogens such as *Cryptosporidium* and *Giardia* and is commonly used for supplementing chlorination and chloramination (Inkinen et al., 2019, 2021). This disinfection method does not produce any odor and has no residual effect for controlling microbial growth in DWDS like chlorination. UV disinfection is not suitable for water with high turbidity.

In addition, traces of heavy metals can enter DWDSs from multiple sources, such as the source water, release from pipe materials, and impurities of chemicals used during DWTP, such as coagulants, flocculants, and disinfectants (Ikonen et al., 2017). Heavy metals together with residual chlorine and possible other antimicrobial agents in DWDS can create a selective ecological pressure for the growth of disinfectant and metal-stress-tolerant microbial communities from wide taxonomic ranges such as bacteria, viruses, and fungi. These stress-bearing recalcitrant microbial groups can also be resistant to other stress factors including antibiotics (Xi et al., 2009; Gullberg et al., 2014; Pal et al., 2015; Chen et al., 2019; Sanganyado and Gwenzi, 2019). Most recalcitrant genes produced by bacteria, viruses, and bacteria due to contact of different antimicrobial agents may carry MGE. These MGE may not only exchange genes between close taxonomic groups such as within same species, family or phylum; but can even exchange between wide taxonomic groups such as bacteria, viruses, and bacteria.

Metal resistance genes (MRGs) such as mercury (stress) resistance genes are common in bacterial communities from wide habitats (Tamar et al., 2003; Hall et al., 2020). Mercury resistance is determined by *mer* operon that can reside on bacterial plasmids, transposons, integrons, and genomic DNA and is frequently linked with ARGs carrying bacteria (Zhang et al., 2018). Furthermore, MRGs can also be mobile and prone to transfer among bacterial communities through HGT (Seiler and Berendonk, 2012; Zhang et al., 2018; Hall et al., 2020).

The emergence of next-generation sequencing (NGS) technologies has provided an unprecedented opportunity to enhance our perspective of the microbial ecology in drinking water systems (Gomez-Alvarez et al., 2014, 2015; Inkinen et al., 2019, 2021; Tiwari et al., 2021). The current study utilized NGS-based high-throughput metagenomic shotgun sequencing for the characterization of bacterial resistome in five DWDSs with different water sources and treatment processes in Finland. This exploratory study aimed to understand the effect of source water and disinfection strategy on the prevalence of ARGs and MRGs in DWDSs.

## Materials and Methods

### Water Sampling

A total of ten water samples, two samples from each five DWDSs (A–E) in Finland were collected on different weeks during August–September 2015 (Table 1). These distribution systems produced high-quality drinking water from different raw water sources and treatment methods. DWDS A and B are located in the same city and use artificially recharged groundwater (ARGW) as source water without the addition of disinfectant during treatment. DWDS C and D use surface water and DWDS E use groundwater as source water. The water in DWDS C and E is treated with chlorine, while DWDS D used chloramine as a disinfectant, and all three locations use UV treatment.

**TABLE 1:** Characteristics of drinking water distribution systems (DWDS) included in the study (Ikonen et al., 2017; Inkinen et al., 2019, 2021).

DWDS	Raw water	Production capacity (m^3^/day)	Treatment process	Disinfection strategy
A	ARGW	6 300	Aeration, lime stabilization, flocculation, clarification, addition of sulfuric acid, and sand filtration	No disinfection
B	ARGW	2 300	Aeration, lime stabilization, flocculation, clarification, and sand filtration	No disinfection
C	Surface water	40 000	Ferric sulfate coagulation, flotation, sand filtration, and activated carbon filtration	UV light, ClO_2_, Cl
D	Surface water	126 000	Ferric sulfate coagulation, clarification, sand filtration, ozonation, and activated carbon filtration	UV light, NH_2_Cl
E	Groundwater	3 000	Aeration, limestone filtration	UV light, NaOCl

*DWDS, drinking water distribution system; ARGW, Artificially recharged groundwater.*

A dead-end ultrafiltration method with REXEED-25A (Asahi Kasei Medical Co., Ltd., Tokyo, Japan) ultrafiltration capsules were used to concentrate a volume of 100 L as described in Inkinen et al. (2019, 2021). Water samples from chlorinated waterworks were treated with a 1% solution of sodium thiosulphate (18 mg/ml Na_2_S_2_O_3_.5H_2_O) to deactivate residual chlorine. Cells were eluted from capsules to 500 ml of backflush solution containing 0.01% sodium polyphosphate, 0.001% Antifoam Y, and 0.5% Tween-80 in sterile water. A secondary concentration for dead-end ultrafiltration eluates was performed by filtrating a volume of 350 ml through a 0.22 μm Express PLUS membrane filter (Merck KGaA, Darmsadt, Germany). The membranes were frozen at −75°C or lower before the extraction of nucleic acids.

### Nucleic Acid Extraction and Nuclease Treatments

Nucleic acids (NAs) were extracted from Express PLUS membranes with a Chemagic DNA Plant kit (Perkin Elmer, Waltham, MA, United States), as described earlier Inkinen et al. (2019, 2021). The extraction series included a positive extraction control and a negative process control. DNA concentrations (ng/μl) from extracts were measured with a Qubit minifluorometer using a Qubit dsDNA HS Assay kit (Thermo Fisher Scientific, Waltham, MA, United States). DNA was purified with RNase A (Thermo Fischer Scientific, Waltham, MA, United States) from 15 or 50 μl of extracted nucleic acids, depending on the DNA concentration. DNA samples were sent to GATC BioTech AG (European Genome and Diagnostics Centre, Germany) for shotgun metagenomics sequencing.

### Metagenomic Sequencing and Processing

Paired-end standard genomic libraries were prepared using the Illumina HiSeq in the NovaSeq 6000 S2 PE150 XP sequence mode. Libraries were quality-checked using an Agilent 2100 Bioanalyzer/Advanced Analytical Technologies Fragment Analyzer. Prior to assembly, the 150-nucleotide (nt) pair-end reads were subjected to quality filtering and cleaning from adapters and phiX artifacts (algorithm combines kmer and Phred Q based trimming), error corrected, normalized (≤100 ×), and filtered to a minimum length of 100-nt using the bioinformatics software package BBMap v38.22^1^ with the following parameters: trim to the right end (ktrim = r), kmer length (*k* = 23), at read tips search kmers down to this length (mink = 11), hamming distance (hdist = 1), trim adapters based on pair overlap (tbo), trim both reads to the same length (tpe), discard reads with Ns (maxns = 0), trim regions with below quality (trimq = 10), on right end only remove bases with below quality (qtrim = r), discard reads with average quality (maq = 12), discard short reads (minlength = 100), error-correction with BBMerge (ecco = t), error-correction with Clumpify (eccc = t), error-correction with Tadpole (ecct = t), and normalization depth (target = 100). The libraries contained an average (± SD) of 35,428,802 ± 3,956,065 reads per sample.

### Antibiotic and Metal Resistance Gene Analysis

Processed reads were queried for antibiotic resistance genes (ARGs) using the online DeepARG pipeline version 1 (Arango-Argoty et al., 2018) with default settings for alignment (50% minimum identity, *E*-value cutoff of 1e–10), coverage (5% minimum), and a minimum probability of 0.8. Results of annotated ARGs were obtained as an absolute abundance of the ARGs. To compare the ARG content from different samples, the relative abundance of ARGs was normalized to the 16S rRNA content in the sample (Arango-Argoty et al., 2016). ARG “abundance” was expressed as “copy of ARG per copy of 16S-rRNA gene” (thereafter called “ratio”) and is described by Li B. et al. (2015).

Before the analysis of MRG-like sequences, each metagenome sequencing dataset was *de novo* assembled using the software program MEGAHIT v1.2.9 (Li D. et al., 2015) with –min-contig-len 1500 and –k-list 21, 29, 39, 59, 79, 99, 119, and 141. Assemblies (i.e., contigs) were queried for MRGs using the software program AMRFinderPlus v 3.10.1 (Feldgarden et al., 2021) with default parameters. The coverage of gene encoding metal resistance was calculated by mapping metagenomic reads to the contigs using the BBwrap script from the BBMap bioinformatics package (see text footnote 1).

Percentage analysis (SIMPER) was used to determine the percentage contribution of AR to the differences observed between sites (Clarke, 1993). Non-metric multidimensional scaling (nMDS), based on the Bray-Curtis distance obtained from the relative abundance of each ARG, was used to describe the relationships among samples. Diversity indices and nMDS plots were generated with the software program PAST v4.06 (Hammer et al., 2001).

## Results

### Resistance Gene Prevalence in Drinking Water Distribution Systems

**FIGURE 1:**
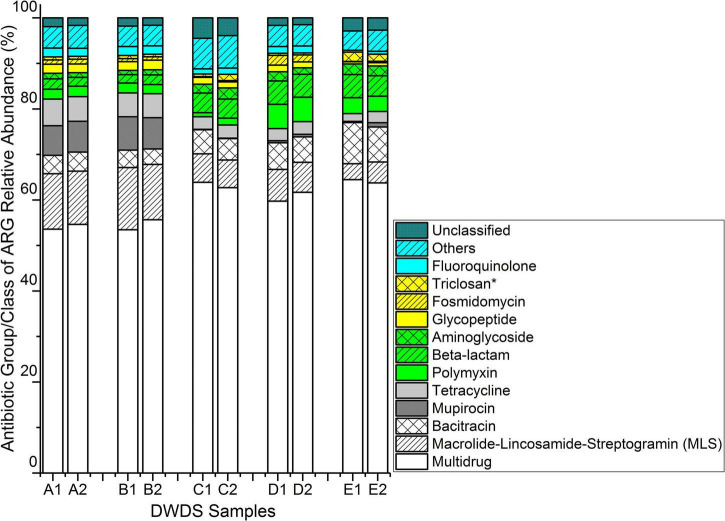
Relative abundance of ARGs conferring resistance to different antibiotic classes/groups obtained from five drinking water distribution systems (DWDSs A–E) using high-throughput metagenome sequencing of total nucleic acids. *Triclosan is an antimicrobial agent, not an antibiotic. ARG having a relative abundance of less than two percent in all samples were grouped into “Others.” ARG types categorized as unclassified were the class/type of these ARGs were not identified with DeepARG.

A total of 430 ARGs from all water samples were identified with an average of 294, 296, 225, 272, and 232 ARGs for two samples collected in consecutive weeks at DWDSs A to E, respectively. The data were normalized to 25,000 read counts in each sample. Among them, 140 ARGs were common in all DWDSs. The ARG diversity (Shannon index) of antibiotic resistomes was relatively lower in DWDSs with no disinfectant. However, the number of total gene types was higher (median: 295, range from 289 to 301, *n* = 4) in non-disinfected than in chlorine or chloramine disinfected DWDSs (median: 214, range from 214 to 273, *n* = 6) (Supplementary Figure 2). Genes with a relative abundance greater than two percent were found to confer resistance to ten different classes of antibiotics, namely macrolide-lincosamide-streptogramin (MLS), bacitracin, mupirocin, tetracycline, polymyxin, beta-lactam, aminoglycoside, glycopeptide, fosmidomycin, and fluoroquinolone (Figure 1). Furthermore, genes associated with multidrug resistance and resistance to triclosan (an agent with antimicrobial properties) also had equal abundance.

**FIGURE 2:**
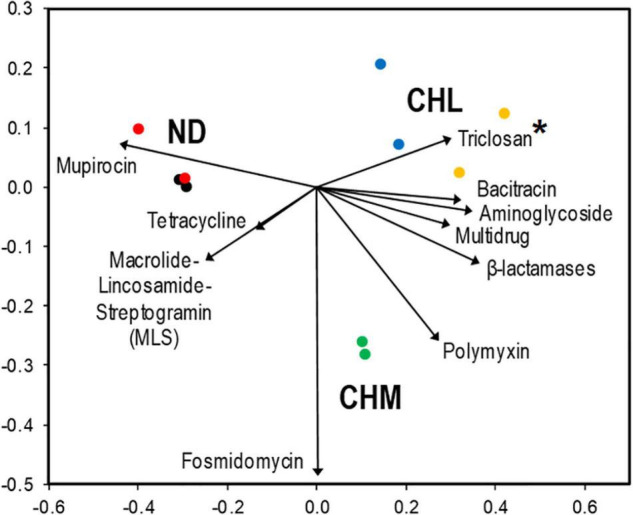
nMDS ordination plot based on the composition of antibiotic resistance genes obtained from DWDS communities. The contribution of ARG categories that explained ∼93% (SIMPER analysis) of the dissimilarity within all samples is represented by the size and direction of vectors. Disinfectant: no disinfection (ND), chlorine (CHL), and chloramine (CHM). Sites: DWDS A (•), DWDS B (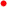), DWDS C (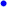), DWDS D (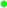), and DWDS E (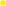). *Genes conferring triclosan, an antimicrobial agent, are also included in this analysis. Stress value: 0.021; Coordinate 1: 67.8%; and Coordinate 2: 10.1%.

The multidrug resistance gene group had the highest relative abundance of reads in all DWDSs. It ranged from 53.5% in DWDS B sample 1 to 64.5% in DWDS E sample 1 (Figure 1). The nMDS ordination analysis revealed that genes associated with multidrug resistance were more abundant in DWDSs using disinfection than in DWDSs without disinfection (Figure 2). Most of the genes and sequence reads identified in this study were associated with multidrug resistance. For instance, DWDS E sample 1 was comprised of 72 genes and DWDS B sample 2 was comprised of 99 genes associated with multidrug resistance (Supplementary Table 1 and Supplementary Figure 2). The majority (>80%) of reads in the multidrug resistance gene group were represented by the genes *rpoB2, Multidrug ABC transporter, mexF*, and *ompR* (Supplementary Figure 3).

Macrolide-lincosamide-streptogramin resistance genes had the second-highest relative abundance, ranging from 3.5% in DWDS E sample 1 to 13.7% in DWDS B sample 1 (Figure 1). The nMDS ordination analysis revealed that the MLS was more related to drinking water systems with non-disinfected treatments (Figure 2). The highest number of resistance genes belonging to MLS was identified in DWDS B sample 1 (i.e., 37), and the lowest (i.e., 21) was identified in DWDS C sample 1 (Supplementary Figure 2). Resistance genes of the MLS group, which includes *macB, llmA 23S rRNA methyltransferase, macA, vgaC*, and *carA*, represented the majority (>80%) of reads (Supplementary Figure 4).

Bacitracin resistance genes were the third most abundant, with a relative abundance ranging from 3.4% in DWDS B sample 2 to 9.1% in DWDS E sample 1 (Figure 1). This group is composed of only two genes, *bacA* and *bcrA* (Supplementary Figure 5). The nMDS ordination analysis revealed that bacitracin resistance genes were more related to DWDS with chlorine as a disinfectant (Figure 2). Only one type of mupirocin resistance gene, *Staphylococcus mupA*, was identified in DWDSs A and B (relative abundance ∼ 7%) but <1% in DWDSs C to E (Figure 1). Tetracycline resistance genes were mostly associated with non-disinfected DWDSs (Figure 2), with a relative abundance of 5–6% in DWDSs A and B and only 2–3% in DWDSs C–E (Figure 1). Our analysis identified a diverse group of tetracycline resistance genes in our samples (Supplementary Figure 6). Beta-lactam resistance genes were relatively more abundant in disinfected DWDS C–E (4–5%) than in non-disinfected DWDS A and B (2%) (Figure 1 and Supplementary Figure 7). The rest of the abundant genes in the studied samples belonged to the antibiotic classes polymyxin, aminoglycoside, glycopeptide, fosmidomycin, and fluoroquinolone, as well as an antimicrobial agent, triclosan (Figure 1 and Supplementary Figures 8–12). The nMDS ordination analysis revealed that aminoglycoside and triclosan-related genes were related to disinfected distribution systems, and polymyxin and fosmidomycin resistance genes were specifically related to the chloraminated distribution system D (Figure 2). ARGs that were conferring resistance to antibiotic groups other than those mentioned above had an abundance lower than 2% in all ten samples and were therefore grouped and referred to as “other antibiotic groups” (Figure 1 and Supplementary Figure 13). The distribution of ARGs conferring resistance to antibiotics of unclassified groups in different DWDS samples is shown in Figure 1 and Supplementary Figure 14.

**FIGURE 3:**
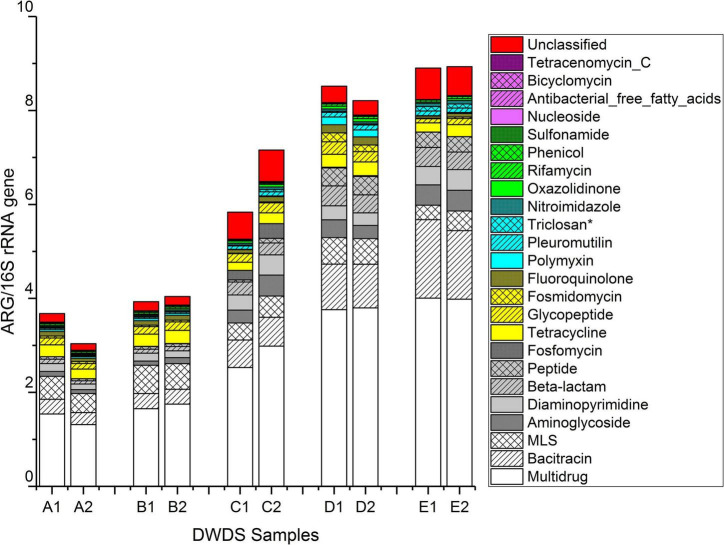
The ratio between ARG reads obtained from metagenomic libraries and the16S rRNA amplicon library. *Genes conferring resistance to triclosan, an antimicrobial agent.

The ratios of ARGs to 16S rRNA gene reads are shown in Figure 3 and Supplementary Figure 15. The total ratio of ARG reads to 16S rRNA gene reads ranged from 3.0 in DWDS A sample 2 to 8.9 in DWDS E sample 2 (Figure 3). On average, the ratio in DWDS A was 3.4, DWDS B was 4.0, DWDS C was 6.5, DWDS D was 8.4, and DWDS E was 8.9. Overall, the ratio of ARG reads to 16S rRNA gene reads were 2.1 and 2.3 times higher in chlorinated DWDSs (C and E) and chloraminated DWDS (D), respectively, than in the non-disinfected DWDSs (A and B).

### Metal Resistance Genes in Drinking Water Distribution Systems

**FIGURE 4:**
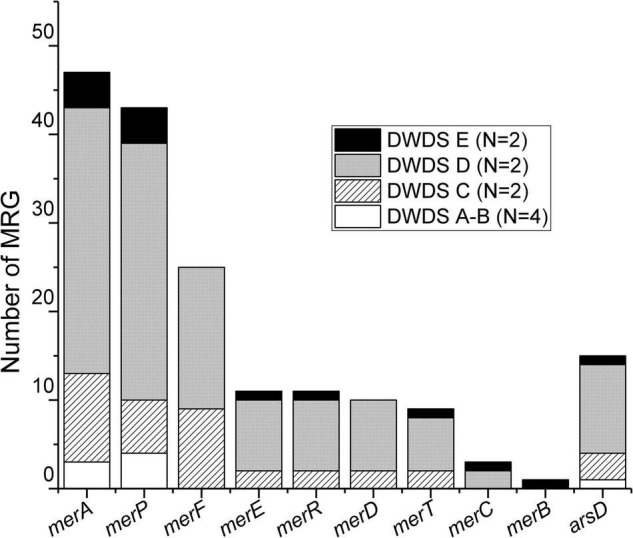
Distribution of MRGs in various DWDSs (A–E). DWDS A and B are pooled together due to the presence of a low number of MRGs in these samples. N, number of samples.

Our analysis identified 177 metal (stress) resistance gene (MRG) sequences in assemblies generated from DWDS metagenomes (Supplementary Table 2). Most of the sequences recovered were associated with mercury and a few with arsenic resistance with a high number of sequences affiliated with disinfected DWDSs of surface waterworks (C: 20.3% and D: 66.1%). The abundance decreased in disinfected groundwater (E: 7.9%) and in non-disinfected DWDSs A and B to 2.3% and 3.4%, respectively. MRGs were associated with mercury (*merA*, *merB*, *merC*, *merD*, *merE*, *merF*, *merP*, *merR*, and *merT*) and arsenic (*arsD*) resistance genes (Figure 4). The metagenomes from DWDS A contained only four MRG sequence reads, annotated as the mercury resistance *merP* gene (Supplementary Table 2). Six MRG sequences were identified in DWDS B samples and were identified as arsenic stress gene *arsD* and mercury stress genes *merA* and *merP* (Supplementary Table 2). In DWDS C, a total of 36 MRG sequences were annotated with *merA*, *merC*, *merD*, *merE*, *merF*, *merP*, *merR*, *merT*, and *arsD* genes (Supplementary Table 2). The highest number of recovered MRG sequences (117) were detected from DWDS D, and the most common MRGs were *merA, merF*, and *merP* (Supplementary Table 2). The samples from DWDS E were represented with a total of 14 MRG sequences, which were annotated as *merA*, *merB*, *merC*, *merE*, *merP*, *merR*, *merT*, and *arsD* genes (Supplementary Table 2).

Alignment of metagenomic libraries against MRG assemblies resulted in 1,108,115 reads (<0.4% of the total reads) mapped to the DWDS samples (Table 2). The contribution of aligned MRG reads (i.e., mapped reads) was higher in samples from disinfected DWDSs. Less than 0.01% of reads in the DWDS A samples were mapped to MRG sequences, while the largest contributor of mapped reads sampled were from DWDS C with 1.52% and 2.10%.

**TABLE 2:** Count abundance and proportion of reads mapped to ARG and MRG present in DWDSs A–E metagenomic libraries obtained with AMRFinderPlus.

DWDS	Sample	Libraries	Mapped	
		Read pairs	Read pairs	Percentage (%)
A	A1	15,271,382	374	0.002
	A2	17,289,502	151	0.001
B	B1	17,338,992	1,627	0.009
	B2	15,010,004	120	0.001
C	C1	13,081,790	275,175	2.103
	C2	15,536,089	235,560	1.516
D	D1	16,186,129	156,119	0.965
	D2	19,391,040	190,127	0.980
E	E1	13,350,213	10,398	0.078
	E2	34,688,870	238,464	0.687
Total		177,144,011	1,108,115	

## Discussion

Our results indicated that disinfection during the drinking water treatment process creates selective ecological pressure for the proliferation of antibiotics and metal stress-resistant bacterial communities in DWDSs. The impact of such selective pressure is consistently supported by previous studies (Shi et al., 2013; Li et al., 2017; Amarasiri et al., 2020). Furthermore, our findings suggest that disinfected water concurrently enriches ARGs and MRGs in DWDSs. Specifically, genes associated with multidrug resistance and resistance to triclosan (an agent with antimicrobial properties) and genes that confer resistance against antibiotic classes bacitracin, beta-lactam, and aminoglycoside could be associated with the use of free chlorine as a disinfectant. However, although genes that confer resistance against multidrug resistance and the antibiotic classes fosmidomycin and polymyxin—and mercury and arsenic resistance genes—were mostly associated with the chloraminated water samples, we also suspect that a major cause for the presence of mercury and arsenic resistance genes in the microbial communities of DWDS D can also be due to its old ferric pipes instead of the use of disinfection.

Chlorination (residual chlorine) reduces microbial activity. It is a common process used as a final step during drinking water treatment to ensure stable water quality during distribution (Inkinen et al., 2019, 2021). Despite the disinfection efforts, a significant number of ARB may survive and proliferate in the DWDS. This argument is supported by the high diversity of ARG in disinfected DWDSs. In environments free from antibiotics and heavy metals, the carriage of ARGs and MRGs can negatively affect the fitness cost of the bacterium (Vogwill and MacLean, 2015). However, the detected prevalence of various ARGs and MRGs suggests that the living conditions in the studied DWDSs are favorable for the growth of ARB. Organic molecules including DNA can be more susceptible to chlorine (Gray et al., 2013). Therefore, it is expected to have reduced extracellular DNA abundance in chlorinated media. Stress-resistant communities (e.g., chlorine-resistant bacteria) may contain multidrug-resistant bacteria and even flourish in disinfected DWDS due to a decrease in ecological competition. For example, after the removal of chlorine susceptible bacteria and immediately after the reduction of the concentration of chlorine, the available niches and nutrients are a plethora for the growth of disinfectant resistance community members. Studies have reported that drinking water chlorination may promote the occurrence of plasmid insertion sequences and integrons involved in the horizontal transfer of ARGs (Shi et al., 2013; Lin et al., 2016; Zhang et al., 2021).

The majority of the ARGs identified in this study were genes associated with multidrug resistance. The rest conferred resistance to several different antibiotic classes and an antimicrobial agent (triclosan). Many of the identified antibiotic classes (i.e., MLS, bacitracin, mupirocin, tetracycline, polymyxin, beta-lactam, aminoglycoside, glycopeptide, fosmidomycin, and fluoroquinolone) are used in industrial agriculture in Finland (Finnish Food Safety Authority, 2019) or are among the most prescribed clinical antibiotics groups in Finland (Supplementary Figure 18; ECDC, 2019). After beta-lactams and aminoglycosides, macrolides are the third major antibiotic class used in Finland (Golkar et al., 2018; FFA, 2021). Triclosan is an antibacterial and antifungal agent commonly used in consumer products such as toothpaste, soaps, and detergents.

Although beta-lactams are widely used in both animal and human medicine, the relative abundance of genes conferring resistance to this antibiotic group was relatively low in the drinking water samples of this study. In theory, the intake of ARB and ARGs through drinking water may transfer genes associated with multidrug resistance or genes conferring resistance to different antibiotic classes to gut microbes through the actions of HGT. As the human gut is nutritious and favorable for the growth of different, even potentially pathogenic bacteria, it can function as a hotspot for the horizontal transfer of ARGs (Lerner et al., 2017). Our finding of the plasmid-mediated *aac*(6′)-ll gene in a DWDS using surface water as source water might indeed have some clinical relevance since this gene is frequently found in human-specific bacterial isolates (Majlesi et al., 2018).

To our knowledge, this is the first study revealing MRGs from DWDSs in Finland, with most of the MRGs related to mercury and arsenic resistance. Mercury resistance *mer*-harboring genes are considered widely distributed in nature (Naguib et al., 2018). It is considered that *mer* operons tend to co-exist with several other resistance determinants (metals, xenobiotics, and antibiotics) (Tamar et al., 2003; Sultan et al., 2020). Further, it can be complicated to decide if such resistance is due to co-selection (co-resistance, cross-resistance). The most widely prevalent mercury resistance genes in our study, *mer*A and *mer*B (*mer* operons), are involved in the detoxification of mercury. The protein encoded by the *mer*A gene is commonly found in the bacterial cytoplasm (Tamar et al., 2003), which reduces the toxicity of the organic form Hg^2+^ to the relatively inert volatile inorganic form Hg^0^ (Tamar et al., 2003; Naguib et al., 2018). The protein encoded by *mer*B gene is responsible for the organomercurial demethylation of organic mercury compounds (Tamar et al., 2003). The *Mer* operon including *mer*A is regulated by two genes, *mer*R and *mer*D. These regulatory genes were detected in high numbers in the disinfected DWDS samples of our study and are known to contribute to the horizontal transfer of mercury resistance genes in bacterial communities (Tamar et al., 2003; Hall et al., 2020). The mercury resistance genes *mer*T, *mer*P, and *mer*A are structural genes that form metal-specific activator-repressors of the operon (Tamar et al., 2003). Furthermore, the most common mercury resistant genes in our study—*mer*T, *mer*P, *mer*C, *mer*E, *mer*F, and *mer*H—express different proteins in bacteria (Naguib et al., 2018). The source of mercury and arsenic in DWDSs may originate from the source water, impurities from chemicals (coagulants, flocculants, and disinfectants) used during the drinking water treatment process. Exposure to environmental stressors, including a relevant concentration of heavy metals, may promote the expression of MRG. These genes are mediated in the plasmid and promote the horizontal transfer of genes. Such genes can be ARGs.

The drinking water quality in Finland is regularly monitored (Council Directive, 2020), and non-compliances of the parametric or indicator values are extremely rare, showing excellent water quality (European Commission, 2016). Ikonen et al. (2017) compared the biological and physicochemical characteristics of water samples from the DWDSs included in the present study and reported parameters within an acceptable range. The physical and chemical characteristics of water can have an important role for bacterial communities in DWDS. Importantly, total assimilable organic carbon (AOC) was significantly higher in waterworks using surface water (DWDSs C & D) than in waterworks using artificial groundwater (DWDSs A &B) and groundwater (DWDS E) (Ikonen et al., 2017). Concentrations of analyzed metal ions (Al, Cu, Fe, and Mn) were low and mostly below the limit of detection in all five DWDSs (Ikonen et al., 2017). In future studies, the relationship between ARGs, and MRGs, with the physical-chemical characteristics can be better established by increasing sample numbers from each DWDS.

The detection of ARGs and MRGs in a well-maintained DWDS may illustrate the ubiquity of these genes in the environment, including man-made water systems. DWDSs are considered pathogen-free but still might contain antibiotic and metal resistant opportunistic pathogens, which might be clinically relevant especially if multidrug-resistant (Amarasiri et al., 2020). The results presented here may indicate that the disinfection treatments often used in the current drinking water treatment processes in Finland and elsewhere could lead to enrichment of ARG and MRG into the microbial communities of the distribution systems. More studies are necessary to investigate the co-occurrence of ARGs and MRGs in clinically relevant bacterial species. The current study increases the understanding of how man-made drinking water infrastructure may have an unexpected influence on the microbial communities by creating selective pressures that may even lead to consequences potentially relevant to public health.

## Data Availability Statement

The data for this study have been deposited in the European Nucleotide Archive (ENA) at EMBL-EBI under accession number PRJEB40814 with the following BioSample numbers: SAMEA7465213 (DWDS A1), SAMEA7465214 (DWDS A2), SAMEA7465217 (DWDS B1), SAMEA7465218 (DWDS B2), SAMEA7465220 (DWDS C1), SAMEA7465221 (DWDS C2), SAMEA7465222 (DWDS D1), SAMEA7465223 (DWDS D2), SAMEA7465226 (DWDS E1), and SAMEA7465226 (DWDS E2).

## Author Contributions

IM, ET, and TP conceptualized, acquired funding, supervised the project, and allocated the resources. SS and A-MH contributed to the sample processing and provided the sample-specific metadata. TP and AK were in charge of preparing and shipping the samples for sequencing. VG-A processed the sequence data and analyzed and visualized the data. AT and VG-A interpreted and further visualized the data. AT, VG-A, AS, and TP drafted the initial version of the manuscript. All authors contributed and approved the final version of the manuscript.

## Conflict of Interest

The authors declare that the research was conducted in the absence of any commercial or financial relationships that could be construed as a potential conflict of interest.

## Publisher’s Note

All claims expressed in this article are solely those of the authors and do not necessarily represent those of their affiliated organizations, or those of the publisher, the editors and the reviewers. Any product that may be evaluated in this article, or claim that may be made by its manufacturer, is not guaranteed or endorsed by the publisher.

## Footnotes

1
http://sourceforge.net/projects/bbmap
