# Supplementary material for: Bacterial Genes Encoding Resistance Against Antibiotics and Metals in Well-Maintained Drinking Water Distribution Systems in Finland
Source: Front Microbiol. 2022 Feb 7;12:803094. doi: 10.3389/fmicb.2021.803094 (PMC8859300; doi:10.3389/fmicb.2021.803094)
Supplement: Supplementary file 1 [file Data_Sheet_1.pdf]

Supplementary Material

Bacterial Genes Encoding Resistance against Antibiotic and Metals in Well-Maintained Drinking Water Distribution Systems in Finland

Ananda Tiwari[1, 2*], Vicente Gomez-Alvarez[3], Sallamaari Siponen[1,4], Anniina Sarekoski[1], Anna-Maria Hokajärvi[1], Ari Kauppinen[1,#], Eila Torvinen[4], Ilkka T. Miettinen[1], and Tarja Pitkänen[1,2]

[1]Expert Microbiology Unit, Finnish Institute for Health and Welfare, Kuopio, Finland.

[2]Department of Food Hygiene and Environmental Health, Faculty of Veterinary Medicine, University of Helsinki, Finland.

[3]Office of Research and Development, U.S. Environmental Protection Agency, Cincinnati, Ohio, USA.

[4]University of Eastern Finland, Department of Environmental and Biological Sciences, Kuopio, Finland.

[#] Current address: Finnish Food Authority, Laboratory and Research Division, Virology Unit, Helsinki, Finland.

***Corresponding author: Ananda Tiwari. E-mail address: ananda.tiwari@thl.fi, Tel.: +358 442535468.**

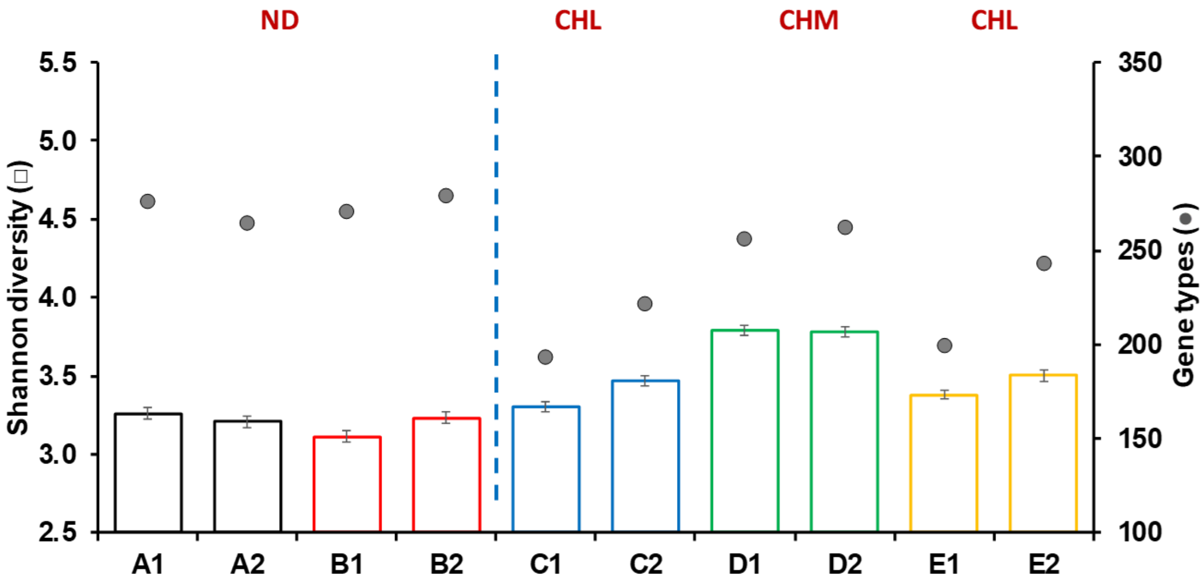

Supplementary Figure S1. Shannon diversity and the number of observed types of antibiotic resistance genes obtained from DWDS communities. Disinfectant: no disinfection (ND), free chlorine (CHL), and chloramine (CHM). Sites: DWDS A (—), DWDS B (—), DWDS C (—), DWDS D (—), and DWDS E (—).

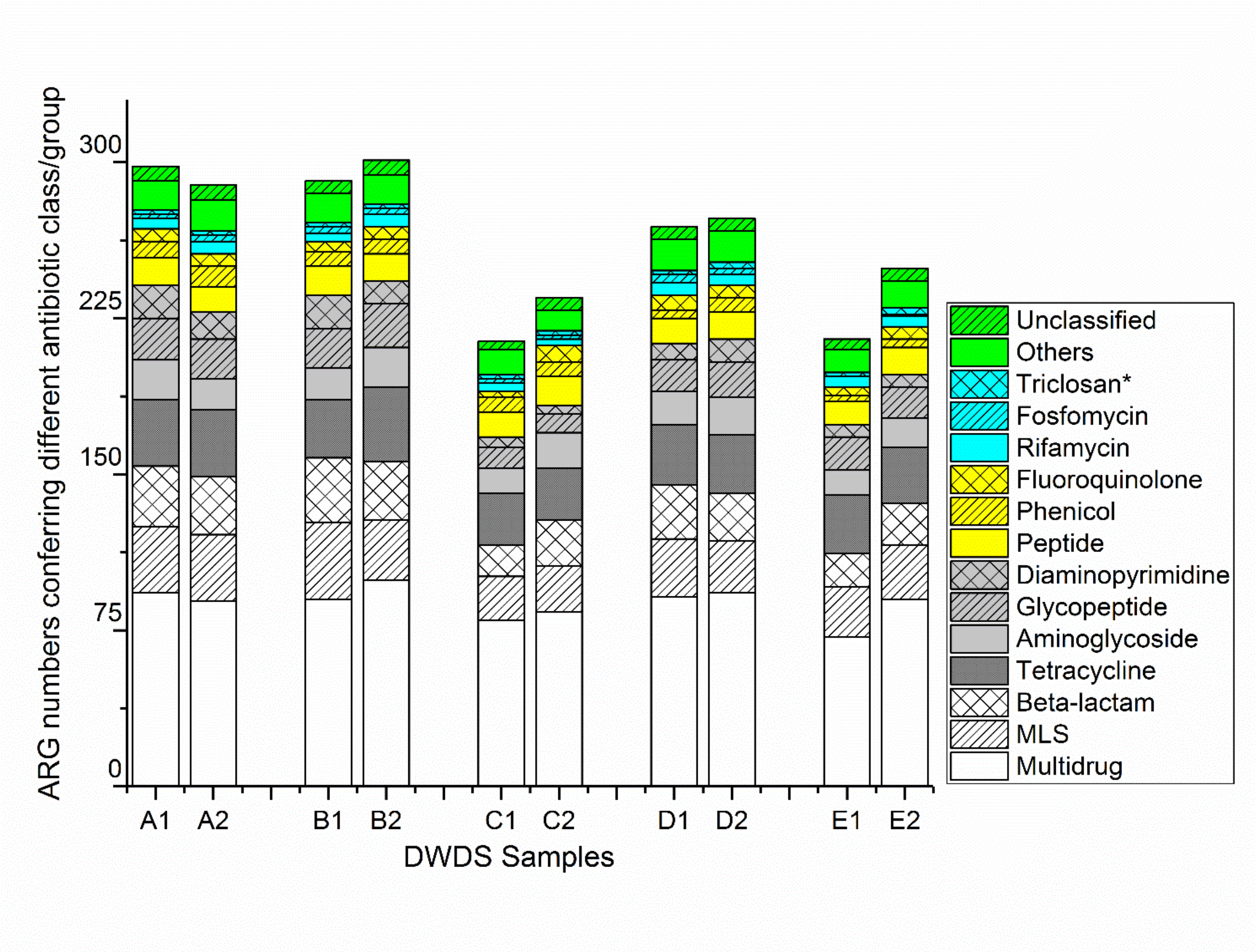

Supplementary Figure S**2.** Total ARG types belonging to different antibiotic classes or groups. All antibiotic classes having less than four genes were grouped into “Others.” Absolute read counts from each DWDS sample are presented in Table S1. *Triclosan is an antimicrobial agent.

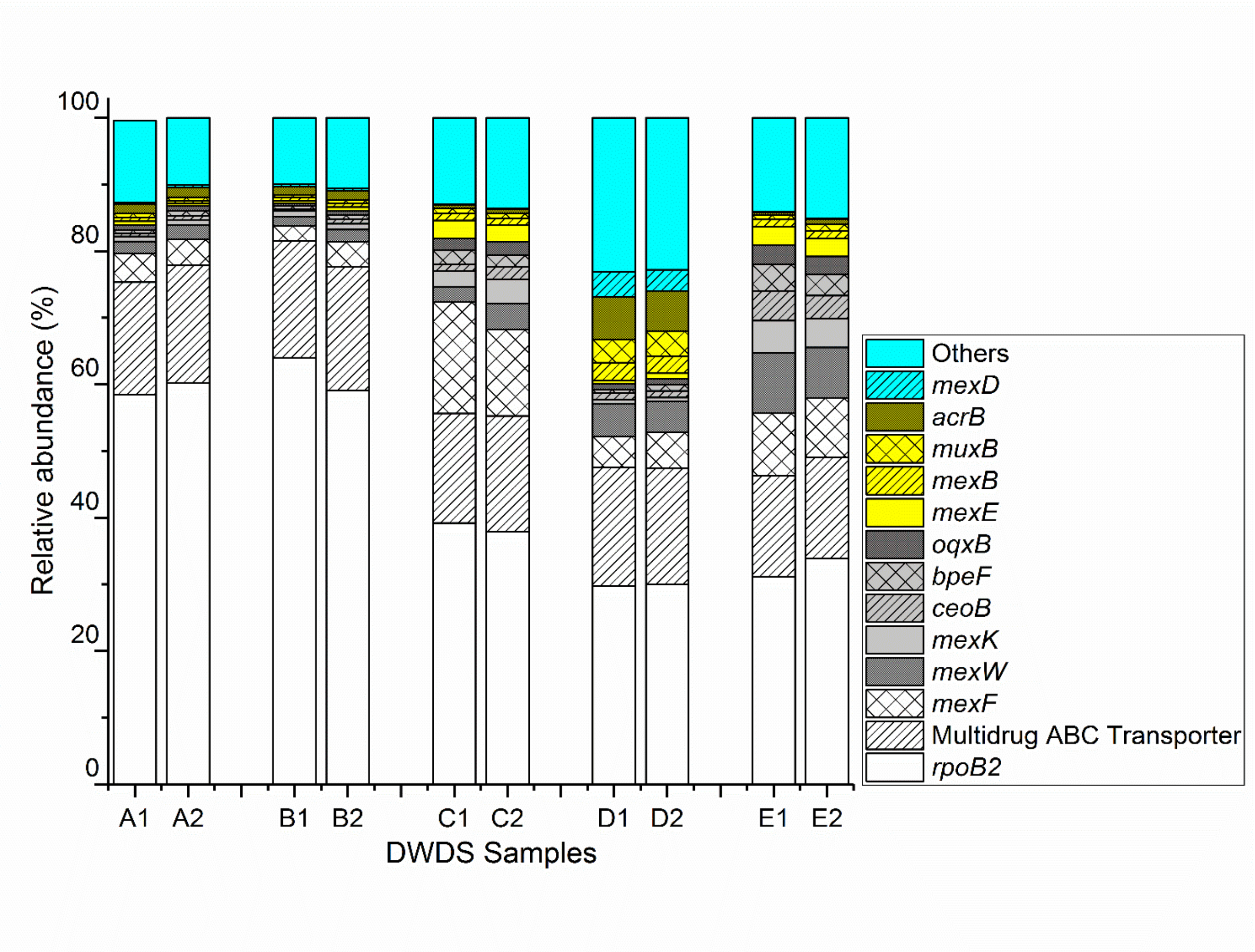

Supplementary Figure S3. Relative abundance of ARGs that confer resistance to multiple drugs in the DWDS samples. All ARG having relative abundance less than two percent in all samples were grouped into "Others." Absolute read counts from each DWDS sample are presented in Table S1.

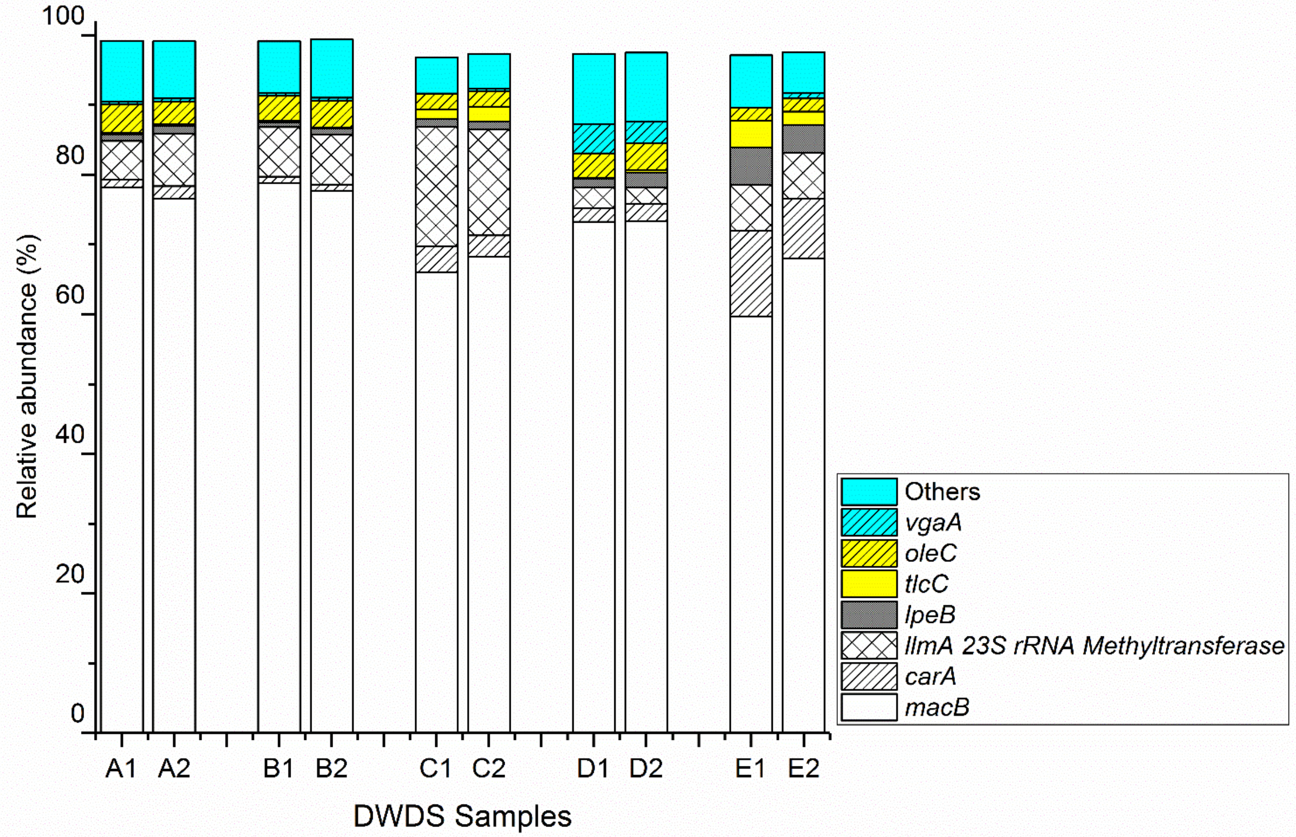

Supplementary Figure S4. Relative abundance of ARGs that confer resistance to Macrolide-Lincosamide-Streptogramin (MLS) antibiotic class in the DWDS samples. All ARG having relative abundance less than two percent in all samples were grouped into “Others.” Absolute read counts from each DWDS sample are presented in Table S1.

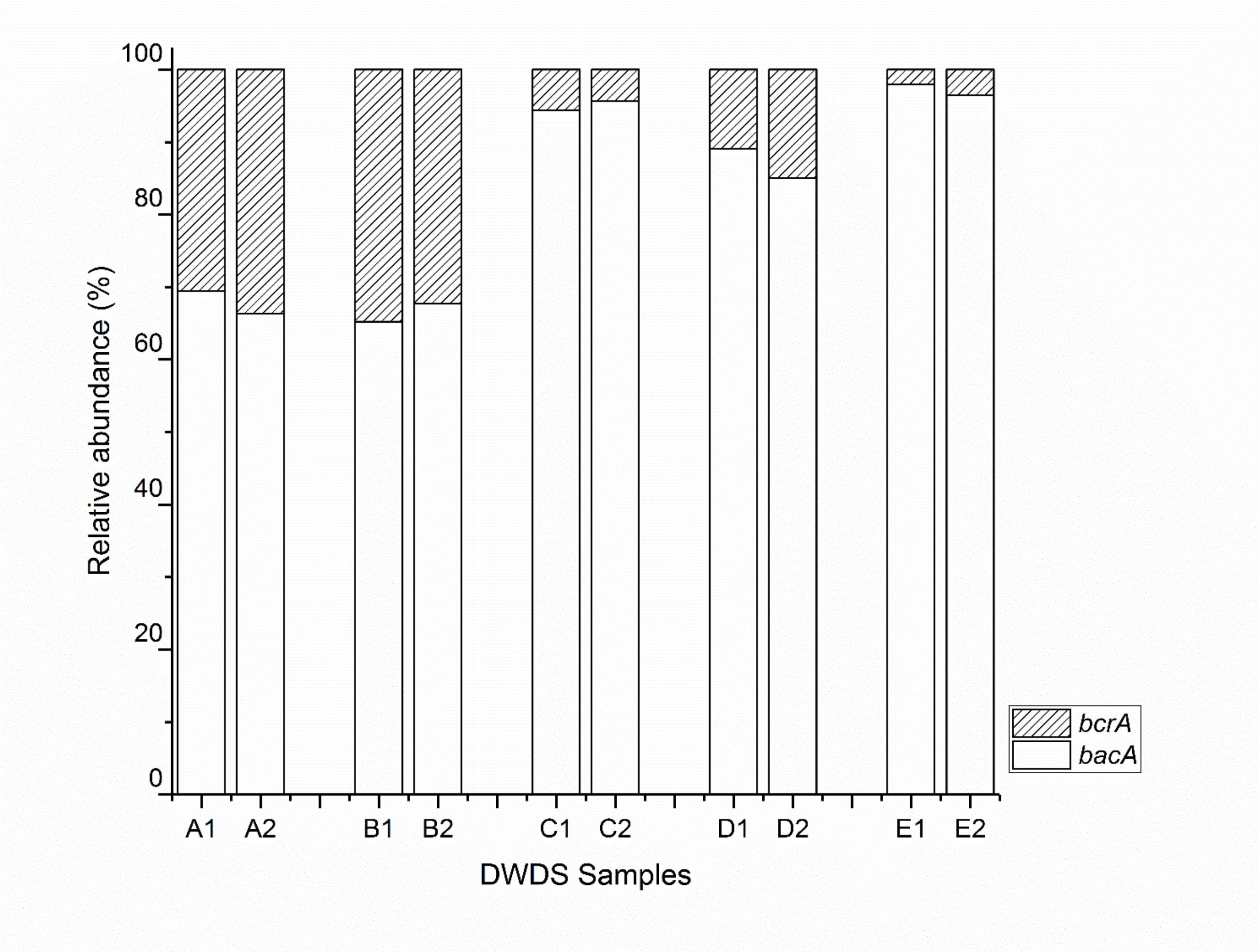

Supplementary Figure S5. Relative abundance of ARGs that confer resistance to bacitracin antibiotic class in various DWDS samples. Absolute read counts from each DWDS sample are presented in Table S1.

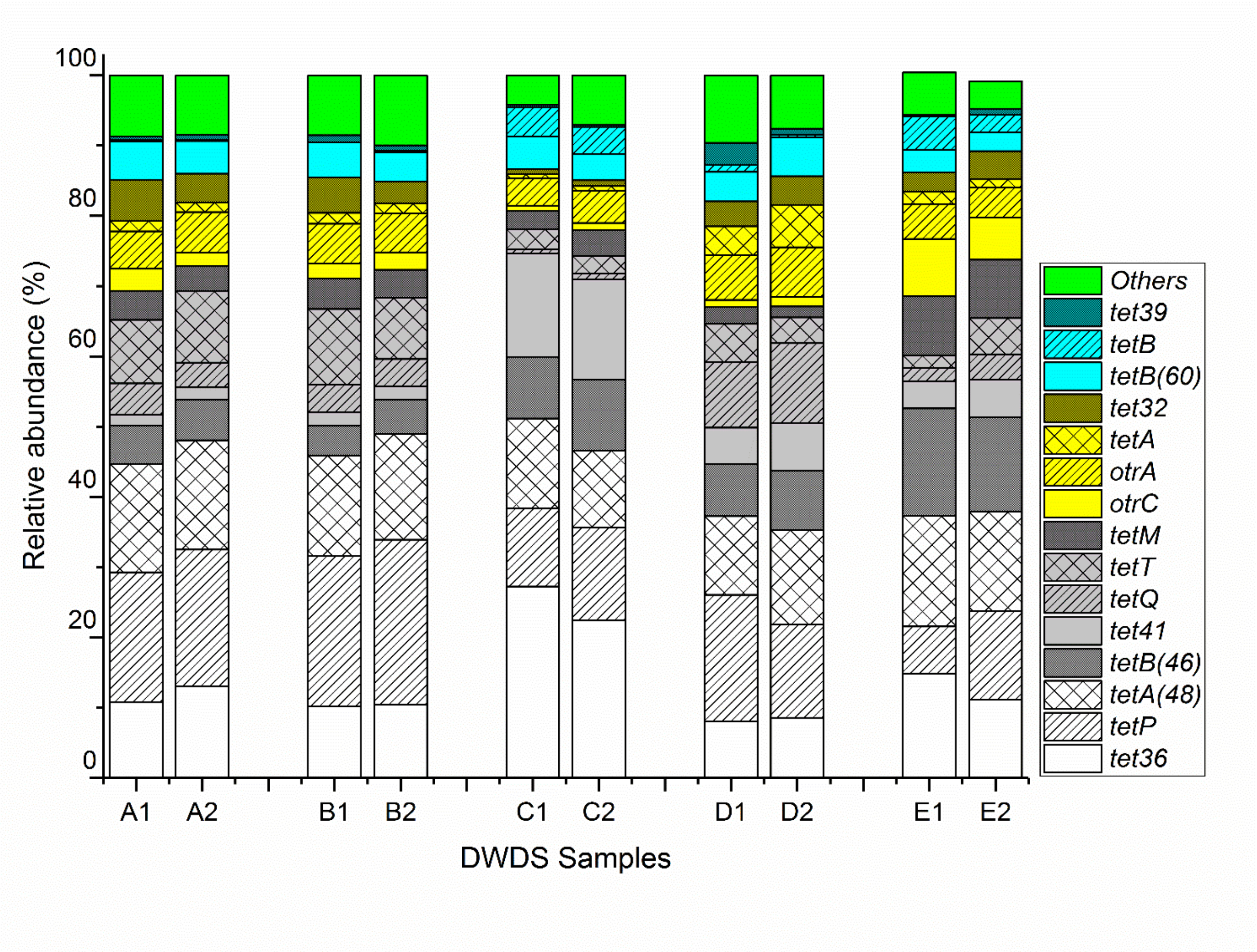

Supplementary Figure S6. Relative abundance of ARGs that confer resistance to tetracycline antibiotic class in various DWDS samples. ARG having a relative abundance of less than two percent in all samples were grouped into “Others.” Absolute read counts from each DWDS sample are presented in Table S1.

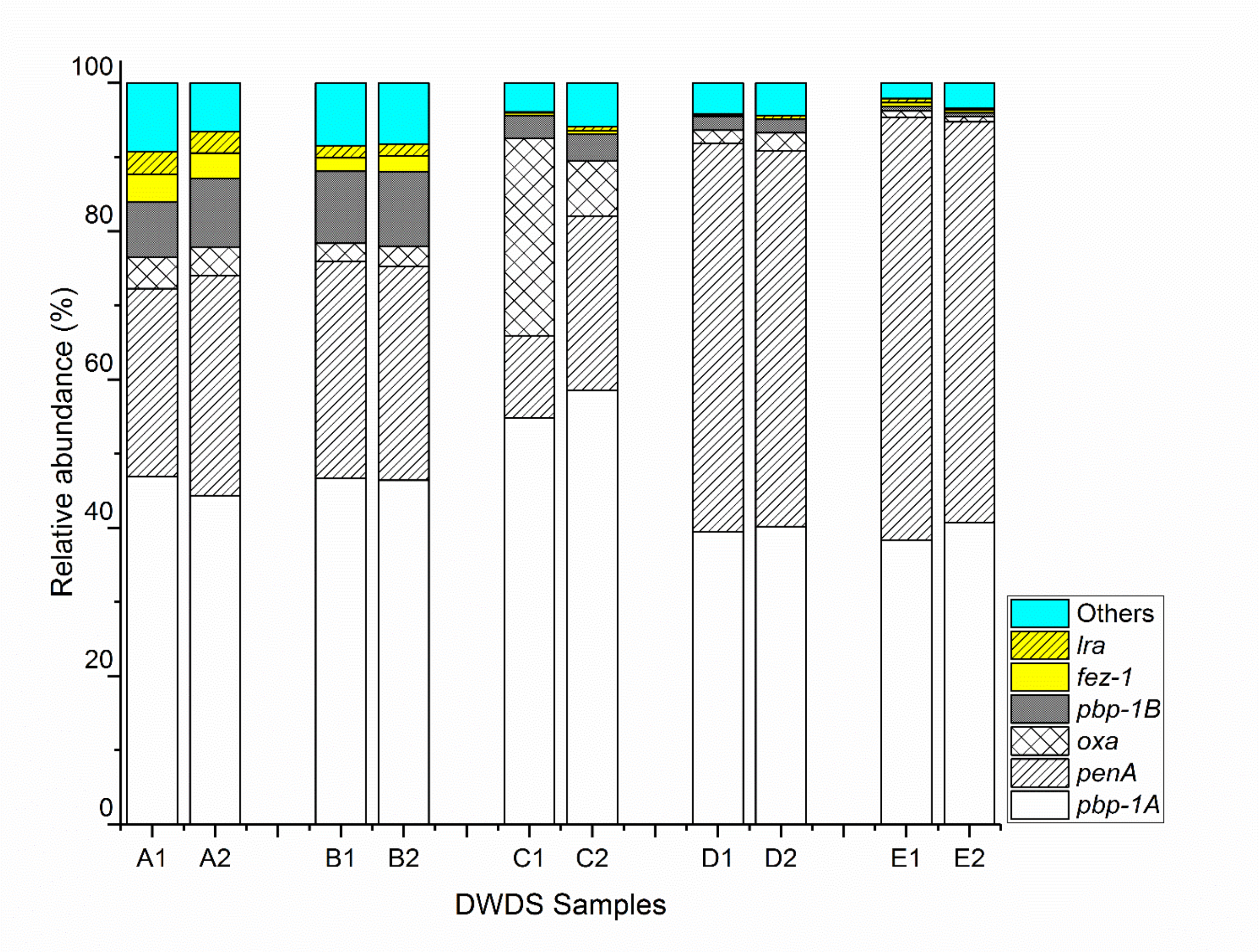

Supplementary Figure S7. Relative abundance of ARGs that confer resistance to beta-lactam antibiotic class in various DWDS samples. ARG having a relative abundance of less than two percent in all samples were grouped into "Others." Absolute read counts from each DWDS sample are presented in Table S1.

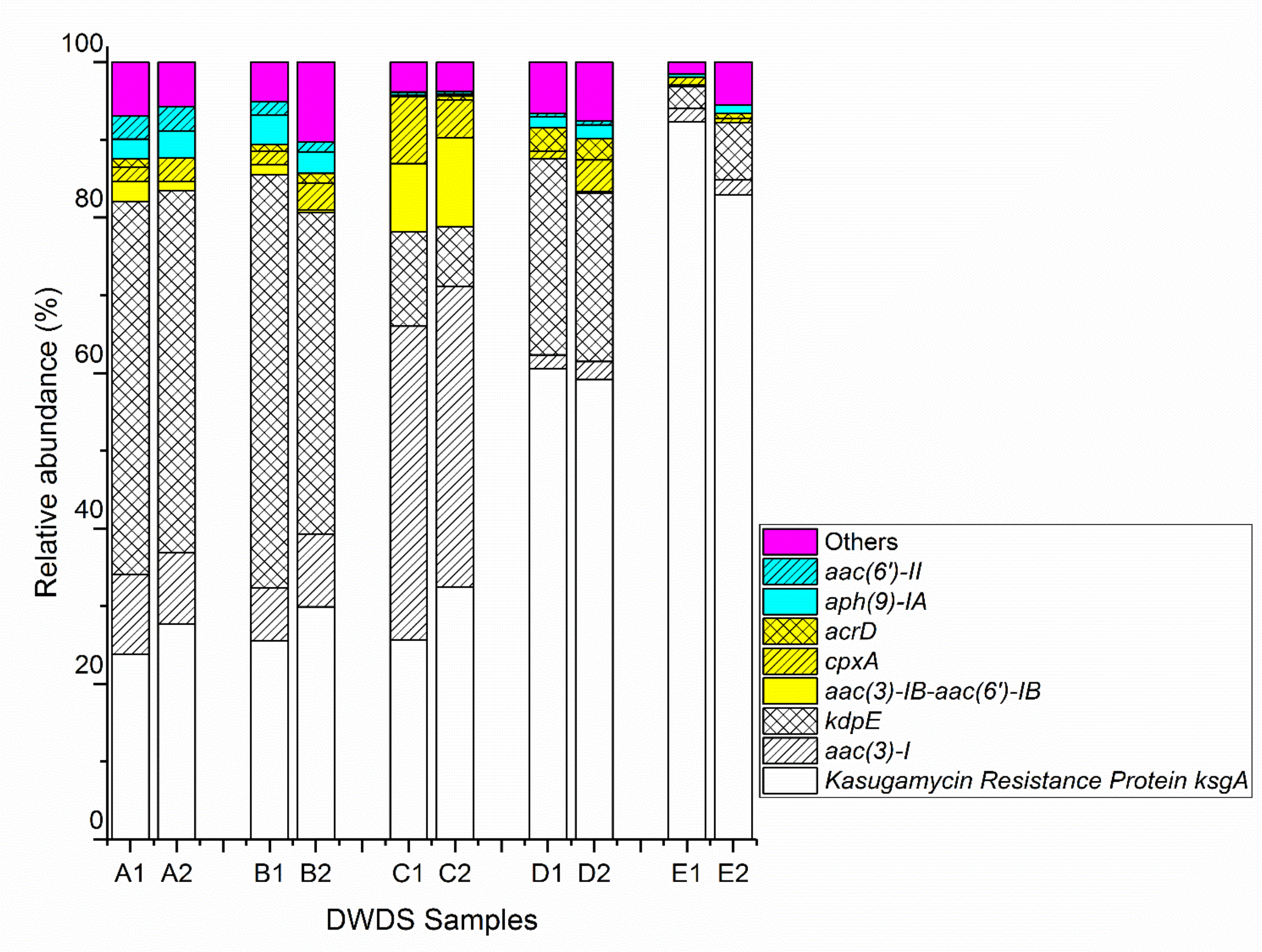

Supplementary Figure S8. Relative abundance of ARGs that confer resistance to aminoglycoside antibiotic class in various DWDS samples. ARG having a relative abundance of less than two percent in all samples were grouped into "Others." Absolute read counts from each DWDS sample are presented in Table S1.

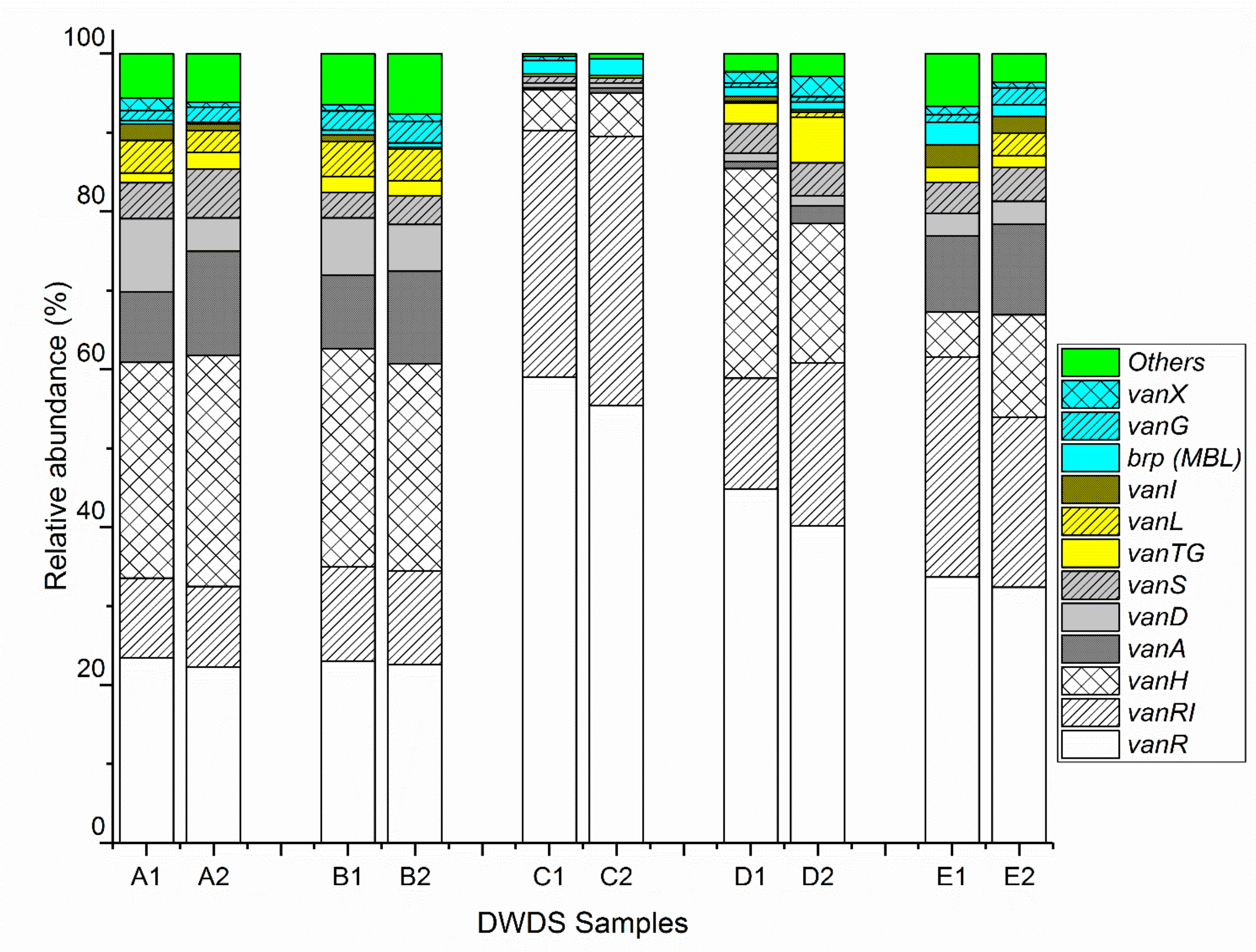

Supplementary Figure S9. Relative abundance of ARGs that confer resistance to glycopeptide antibiotic class in various DWDS samples. ARG having a relative abundance of less than two percent in all samples were grouped into "Others." Absolute read counts from each DWDS sample are presented in Table S1.

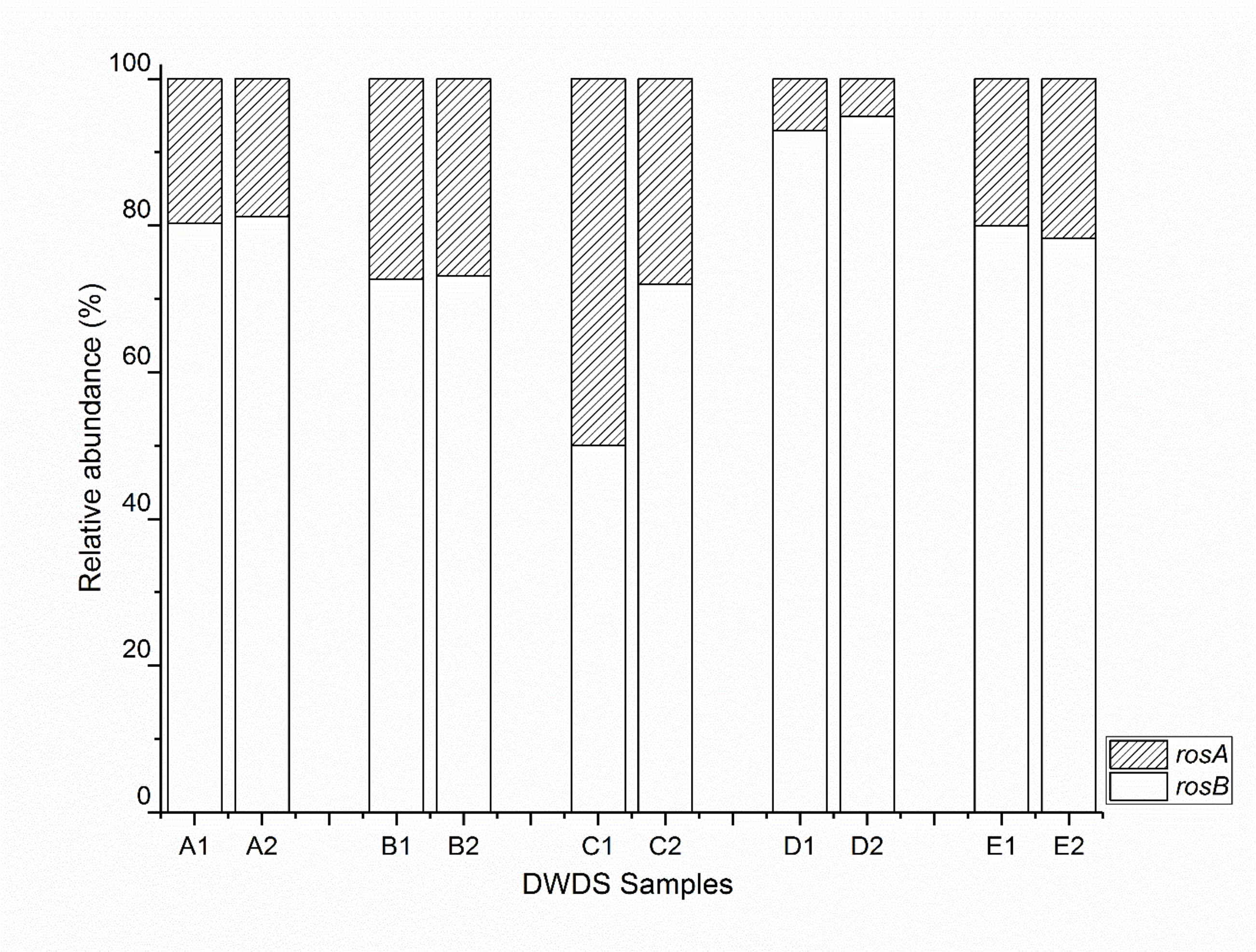

Supplementary Figure S10. Relative abundance of ARGs that confer resistance to the fosmidomycin antibiotic class in various DWDS samples. Absolute read counts from each DWDS sample are presented in Table S1.

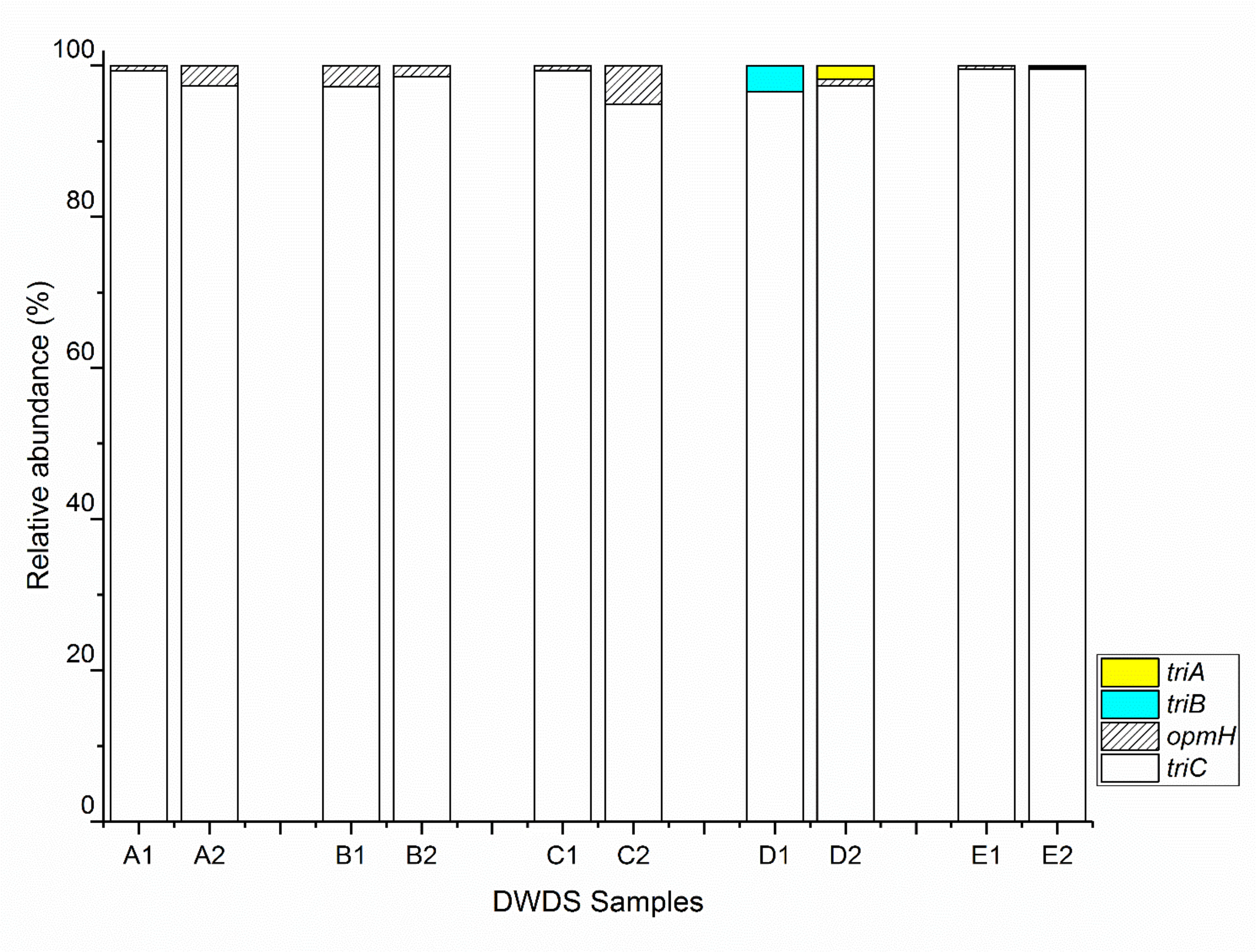

Supplementary Figure S11. Relative abundance of genes that confer resistance to triclosan antimicrobial agent in various DWDS samples. Absolute read counts from each DWDS sample are presented in Table S1.

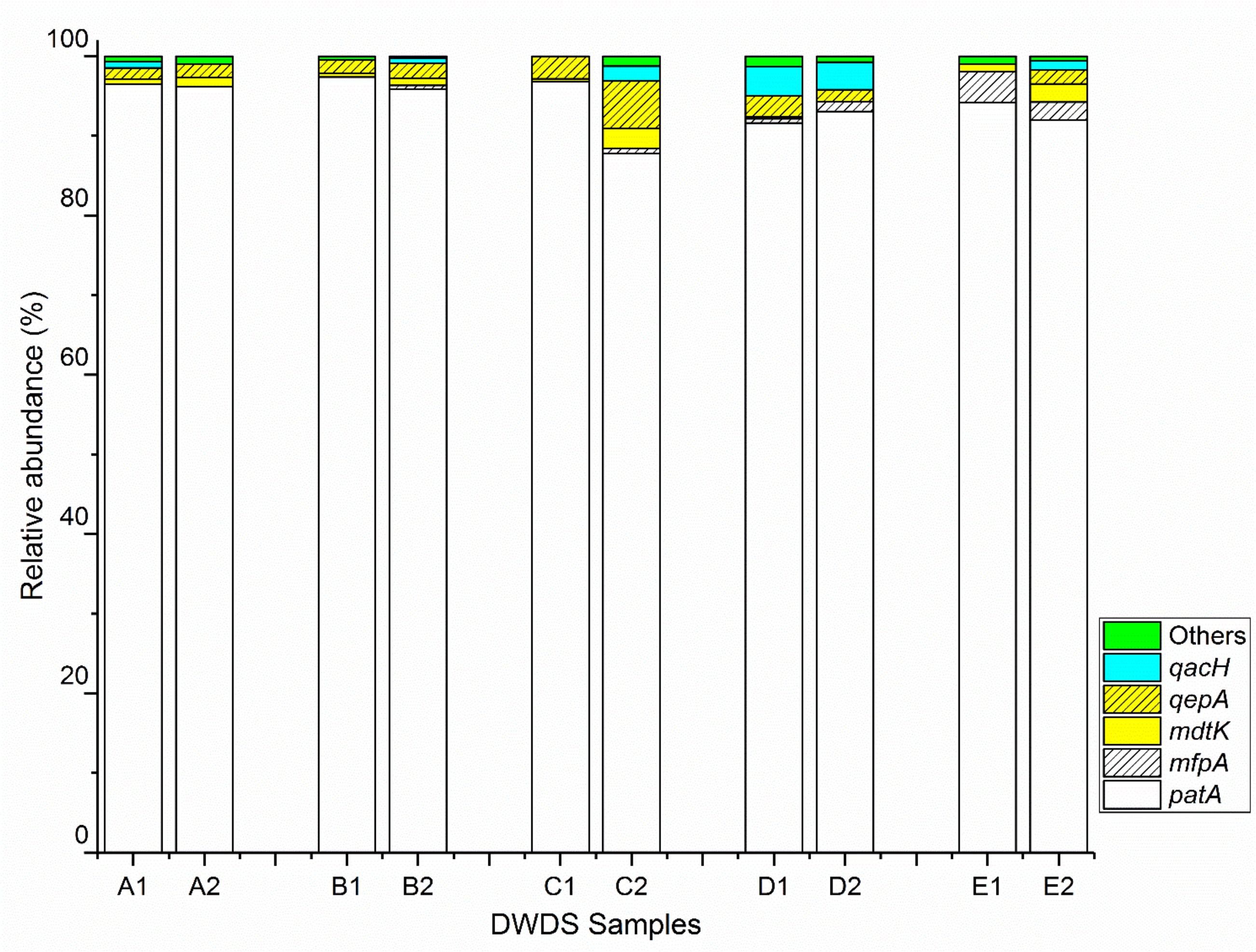

Supplementary Figure S12. Relative abundance of ARGs that confer resistance to fluoroquinolone antibiotic class in various DWDS samples. ARG having a relative abundance of less than two percent in all samples were grouped into "Others." Absolute read counts from each DWDS sample are presented in Table S1.

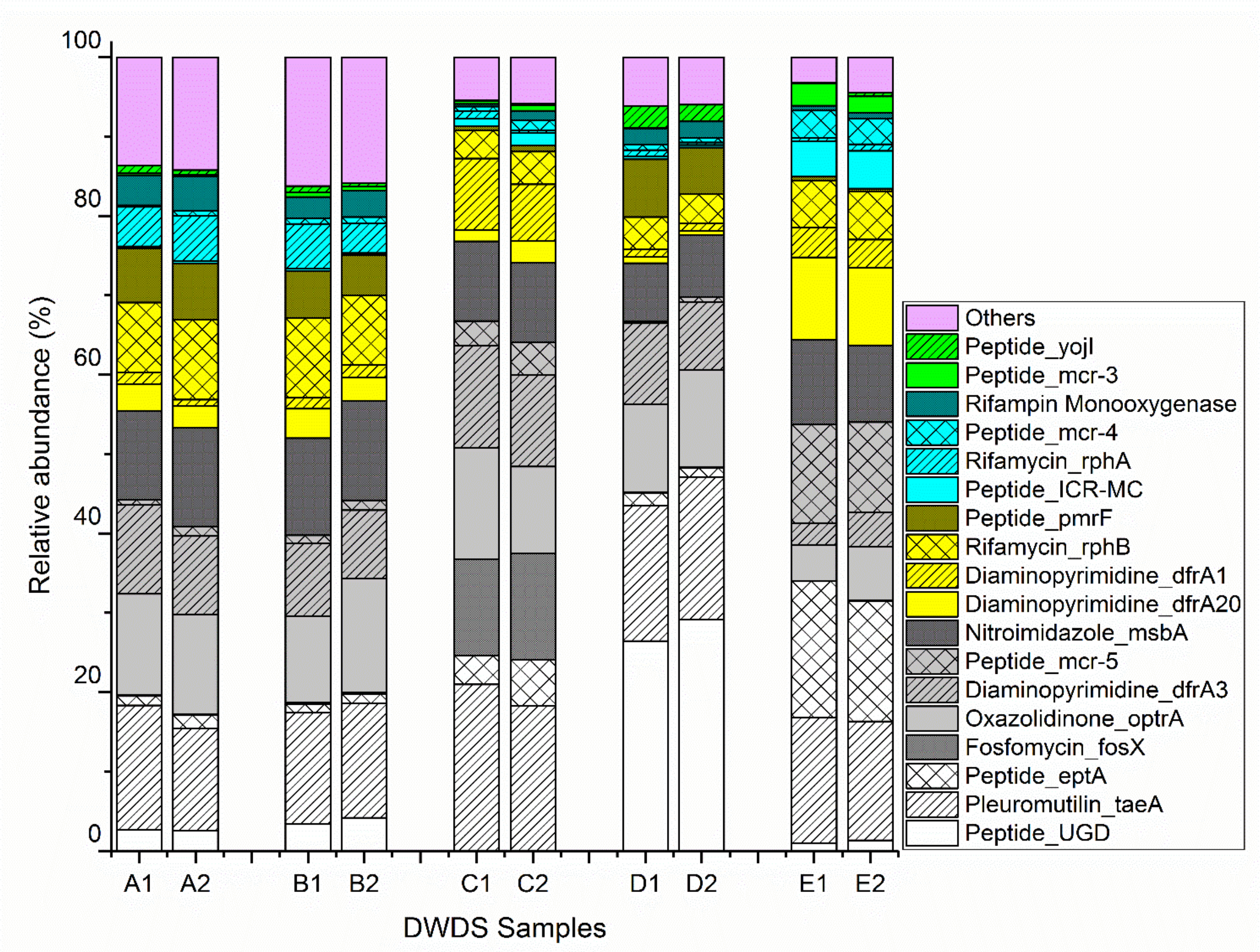

Supplementary Figure S13. Relative abundance of ARGs that confer resistance to other antibiotic classes in various DWDS samples. ARG having a relative abundance of less than two percent in all samples were grouped into "Others." Absolute read counts from each DWDS sample are presented in Table S1.

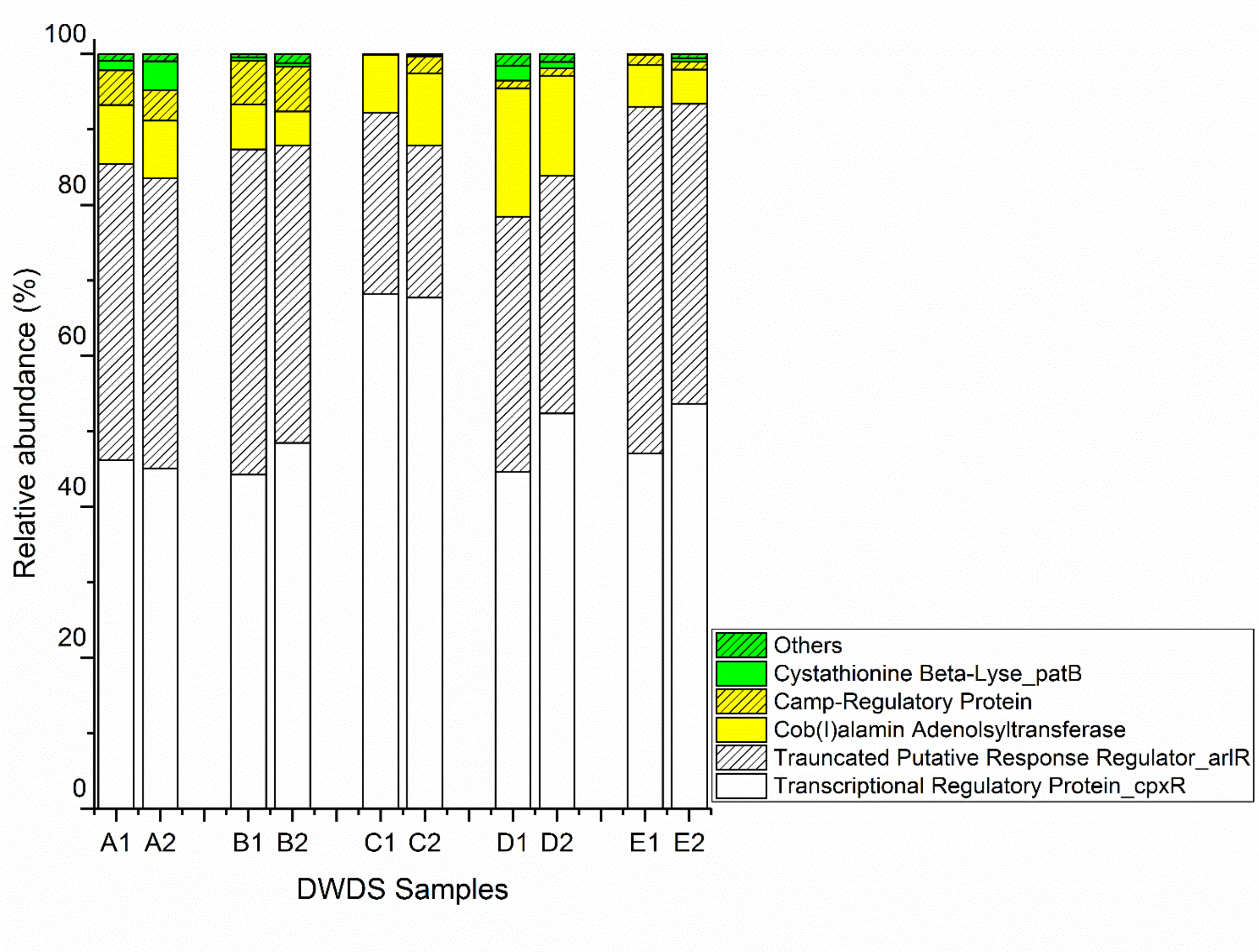

Supplementary Figure S14. Relative abundance of ARGs that confer resistance to unclassified antibiotics in various DWDS samples. ARG having a relative abundance of less than two percent in all samples were grouped into "Others." Absolute read counts from each DWDS sample are presented in Table S1.

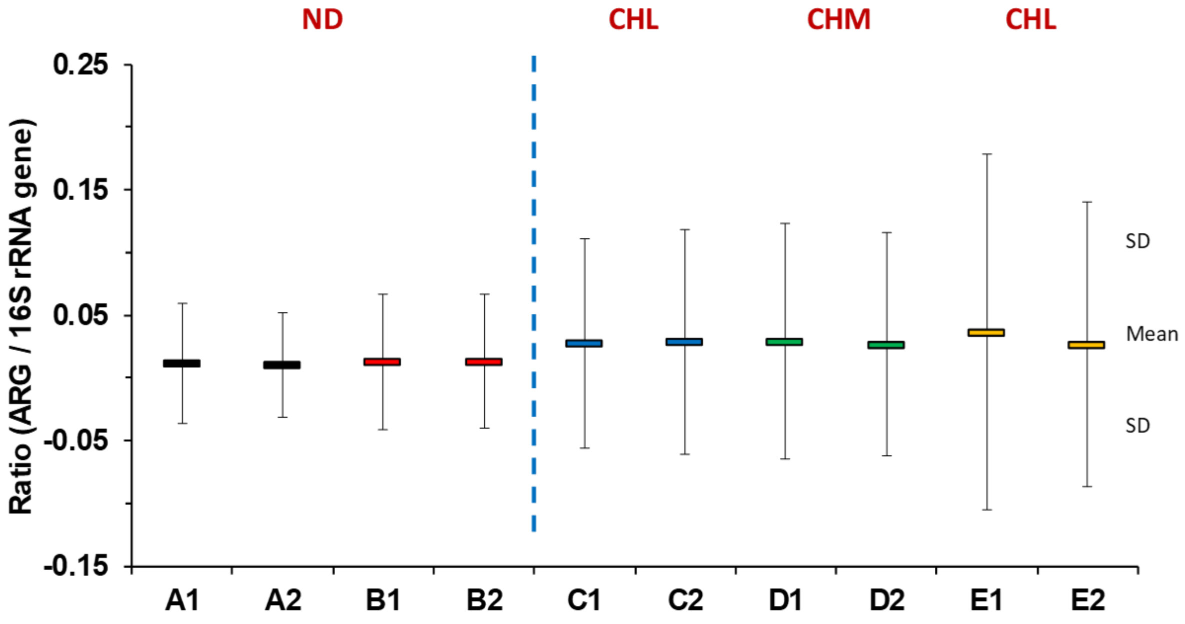

Supplementary Figure S15. Quantitative profile of antibiotic resistance genes obtained from DWDS communities. Disinfectant: no disinfection (ND), free chlorine (CHL), and chloramine (CHM). Sites: DWDS A (—), DWDS B (—), DWDS C (—), DWDS D (—), and DWDS E (—).

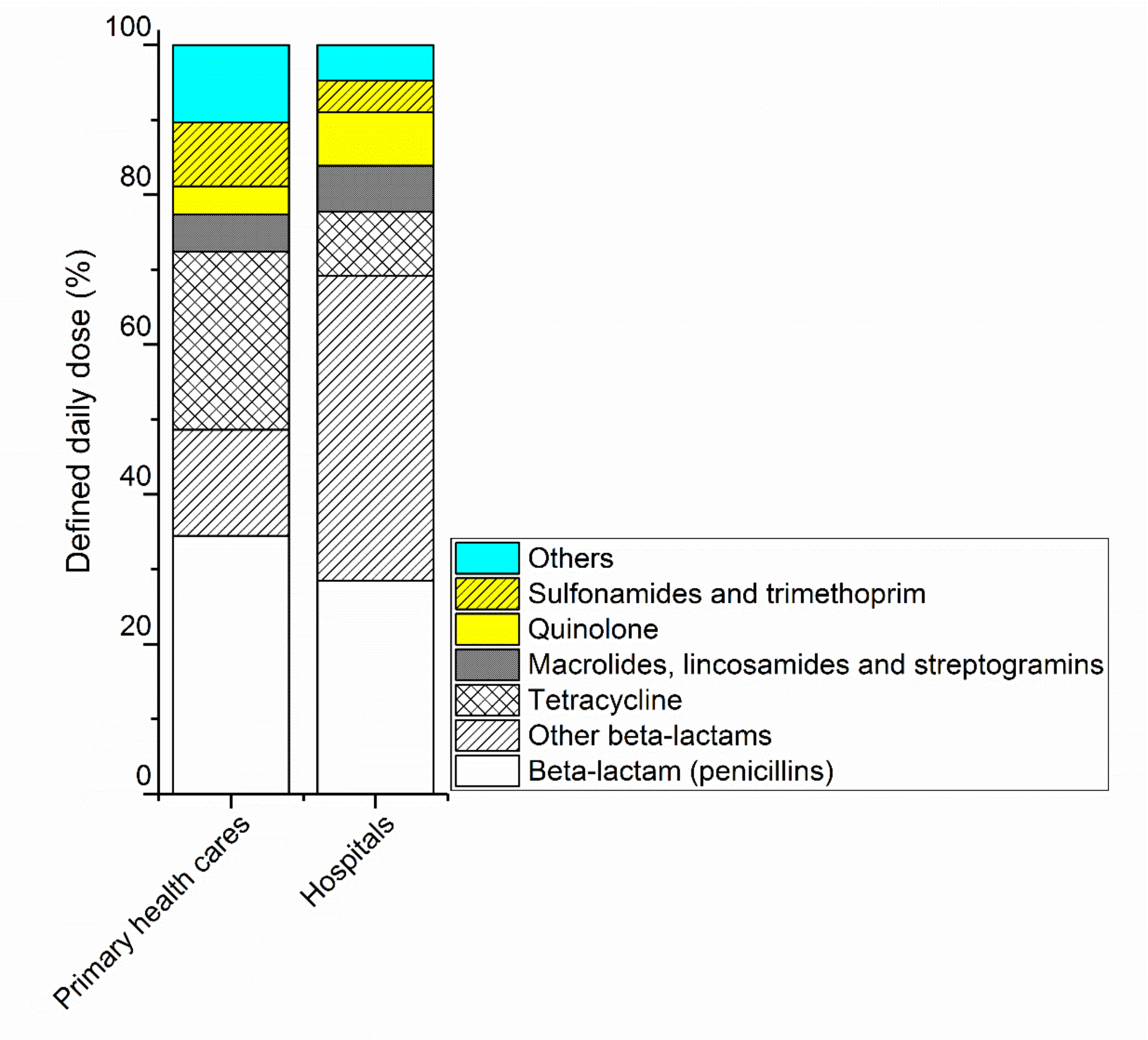

Supplementary Figure S16. The proportion of antibiotics consumption in Finland. Primary health care units prescribe 12.56 defined daily doses per 1000 population, and hospitals prescribe 2.11 defined daily dose per 1000 population in Finland (ECDC, 2019).

Supplementary Table S1. Read counts of ARG classes in DWDSs A–E samples 1 and 2. Data were normalized to 25,000 reads per sample. *Genes from triclosan, an antimicrobial agent, were also included in the analysis.

**Antibiotic groups/ classes**	**DWDS**									
	**A1**	**A2**	**B1**	**B2**	**C1**	**C2**	**D1**	**D2**	**E1**	**E2**
Multidrug	13 764	13 764	13 344	13 721	16 152	15 626	14 922	15 427	16 119	15 868
MLS	3 055	2 952	3 400	3 042	1 520	1 575	1 713	1 675	866	1 177
Bacitracin	1 034	971	975	910	1 324	1 188	1 426	1 382	2 263	1 910
Mupirocin	1 595	1 698	1 805	1 701	22	22	123	141	75	214
Tetracycline	1 373	1 357	1 359	1 377	690	723	698	711	464	602
Polymyxin	311	319	334	286	35	76	555	509	29	58
Beta-lactam	584	519	495	558	1 100	1 003	1 398	1 223	1 249	1 123
Aminoglycoside	273	260	235	321	468	638	499	397	574	527
Glycopeptide	483	471	495	522	349	323	350	311	104	139
Fosmidomycin	244	282	161	186	44	75	512	395	30	83
Triclosan*	145	151	181	136	157	331	117	111	441	406
Fluoroquinolone	481	420	461	465	282	320	382	405	103	174
Others	1 197	1 417	1 319	1 354	1 795	2 084	1 906	1 935	1 937	2 038
Unclassified	461	419	436	421	1 062	1 016	399	378	746	681
Total	25 000	25 000	25 000	25 000	25 000	25 000	25 000	25 000	25 000	25 000

Supplementary Table S2. Heavy metal stress genes identified in the samples originating from DWDSs A–E.

DWDS	Sample	Gene Symbol	Sequence Name	Class	Subclass
A	A1	*MerP*	Mercury resistance system periplasmic binding protein *merP*	Mercury	Mercury
		MerP	Mercury resistance system periplasmic binding protein *merP*	Mercury	Mercury
	A2	*MerP*	Mercury resistance system periplasmic binding protein *merP*	Mercury	Mercury
		MerP	Mercury resistance system periplasmic binding protein *merP*	Mercury	Mercury
B	B1	*ArsD*	Arsenite efflux transporter metallochaperone *arsD*	Arsenic	Arsenite
		MerA	Mercury (II) reductase	Mercury	Mercury
		MerP	Mercury resistance system periplasmic binding protein *merP*	Mercury	Mercury
	B2	*MerA*	Mercury (II) reductase	Mercury	Mercury
		MerA	Mercury (II) reductase	Mercury	Mercury
		MerP	Mercury resistance system periplasmic binding protein *merP*	Mercury	Mercury
C	C1	*MerR*	Mercury resistance transcriptional regulator *merR*	Mercury	Mercury
		MerT	Mercuric transport protein *merT*	Mercury	Mercury
		MerP	Mercury resistance system periplasmic binding protein *merP*	Mercury	Mercury
		MerF	Mercury resistance system transport protein *merF*	Mercury	Mercury
		MerA	Mercury (II) reductase	Mercury	Mercury
		MerD	Mercury resistance co-regulator *merD*	Mercury	Mercury
		MerE	Broad-spectrum Mercury transporter *merE*	Mercury	Mercury
		MerF	Mercury resistance system transport protein *merF*	Mercury	Mercury
		MerA	Mercury (II) reductase	Mercury	Mercury
		MerA	Mercury (II) reductase	Mercury	Mercury
		ArsD	Arsenite efflux transporter metallochaperone *arsD*	Arsenic	Arsenite
		MerF	Mercury resistance system transport protein *merF*	Mercury	Mercury
		MerP	Mercury resistance system periplasmic binding protein *merP*	Mercury	Mercury
		MerA	Mercury (II) reductase	Mercury	Mercury
		MerF	Mercury resistance system transport protein *merF*	Mercury	Mercury
	C2	*MerA*	Mercury (II) reductase	Mercury	Mercury
		MerE	Broad-spectrum Mercury transporter *merE*	Mercury	Mercury
		MerD	Mercury resistance co-regulator *merD*	Mercury	Mercury
		MerP	Mercury resistance system periplasmic binding protein *merP*	Mercury	Mercury
		MerA	Mercury (II) reductase	Mercury	Mercury
		MerP	Mercury resistance system periplasmic binding protein *merP*	Mercury	Mercury

		MerF	Mercury resistance system transport protein *merF*	Mercury	Mercury
		MerA	Mercury (II) reductase	Mercury	Mercury
		MerP	Mercury resistance system periplasmic binding protein *merP*	Mercury	Mercury
		MerF	Mercury resistance system transport protein *merF*	Mercury	Mercury
		MerA	Mercury (II) reductase	Mercury	Mercury
		MerR	Mercury resistance transcriptional regulator *merR*	Mercury	Mercury
		MerT	MerCuric transport protein *merT*	Mercury	Mercury
		MerP	Mercury resistance system periplasmic binding protein *merP*	Mercury	Mercury
		MerF	Mercury resistance system transport protein *merF*	Mercury	Mercury
		MerA	Mercury (II) reductase	Mercury	Mercury
		MerF	Mercury resistance system transport protein *merF*	Mercury	Mercury
		ArsD	Arsenite efflux transporter metallochaperone *arsD*	Arsenic	Arsenite
		MerF	Mercury resistance system transport protein *merF*	Mercury	Mercury
		MerA	Mercury (II) reductase	Mercury	Mercury
		ArsD	Arsenite efflux transporter metallochaperone *arsD*	Arsenic	Arsenite
D	D1	*ArsD*	Arsenite efflux transporter metallochaperone *arsD*	Arsenic	Arsenite
		MerF	Mercury resistance system transport protein *merF*	Mercury	Mercury
		MerF	Mercury resistance system transport protein *merF*	Mercury	Mercury
		MerA	Mercury (II) reductase	Mercury	Mercury
		MerP	Mercury resistance system periplasmic binding protein *merP*	Mercury	Mercury
		ArsD	Arsenite efflux transporter metallochaperone *arsD*	Arsenic	Arsenite
		MerA	Mercury (II) reductase	Mercury	Mercury
		MerD	Mercury resistance co-regulator *merD*	Mercury	Mercury
		MerE	Broad-spectrum Mercury transporter *merE*	Mercury	Mercury
		MerP	Mercury resistance system periplasmic binding protein *merP*	Mercury	Mercury
		MerA	Mercury (II) reductase	Mercury	Mercury
		MerR	Mercury resistance transcriptional regulator *merR*	Mercury	Mercury
		MerT	MerCuric transport protein *merT*	Mercury	Mercury
		MerP	Mercury resistance system periplasmic binding protein *merP*	Mercury	Mercury
		MerC	Organomercurial transporter *merC*	Mercury	Organomercury
		MerA	Mercury (II) reductase	Mercury	Mercury
		MerD	Mercury resistance co-regulator *merD*	Mercury	Mercury
		MerE	Broad-spectrum Mercury transporter *merE*	Mercury	Mercury
		MerF	Mercury resistance system transport protein *merF*	Mercury	Mercury
		MerF	Mercury resistance system transport protein *merF*	Mercury	Mercury
		MerA	Mercury (II) reductase	Mercury	Mercury
		MerP	Mercury resistance system periplasmic binding protein *merP*	Mercury	Mercury
		MerE	Broad-spectrum Mercury transporter *merE*	Mercury	Mercury
		MerD	Mercury resistance co-regulator *merD*	Mercury	Mercury

		MerA	Mercury (II) reductase	Mercury	Mercury
		MerF	Mercury resistance system transport protein *merF*	Mercury	Mercury
		MerP	Mercury resistance system periplasmic binding protein *merP*	Mercury	Mercury
		MerT	MerCuric transport protein *merT*	Mercury	Mercury
		MerR	Mercury resistance transcriptional regulator *merR*	Mercury	Mercury
		MerA	Mercury (II) reductase	Mercury	Mercury
		MerF	Mercury resistance system transport protein *merF*	Mercury	Mercury
		MerP	Mercury resistance system periplasmic binding protein *merP*	Mercury	Mercury
		MerP	Mercury resistance system periplasmic binding protein *merP*	Mercury	Mercury
		MerR	Mercury resistance transcriptional regulator *merR*	Mercury	Mercury
		ArsD	Arsenite efflux transporter metallochaperone *arsD*	Arsenic	Arsenite
		ArsD	Arsenite efflux transporter metallochaperone *arsD*	Arsenic	Arsenite
		MerE	Broad-spectrum Mercury transporter *merE*	Mercury	Mercury
		MerD	Mercury resistance co-regulator *merD*	Mercury	Mercury
		MerA	Mercury (II) reductase	Mercury	Mercury
		MerF	Mercury resistance system transport protein *merF*	Mercury	Mercury
		MerP	Mercury resistance system periplasmic binding protein *merP*	Mercury	Mercury
		MerT	MerCuric transport protein *merT*	Mercury	Mercury
		MerR	Mercury resistance transcriptional regulator *merR*	Mercury	Mercury
		MerP	Mercury resistance system periplasmic binding protein *merP*	Mercury	Mercury
		MerF	Mercury resistance system transport protein *merF*	Mercury	Mercury
		MerA	Mercury (II) reductase	Mercury	Mercury
		MerP	Mercury resistance system periplasmic binding protein *merP*	Mercury	Mercury
		MerP	Mercury resistance system periplasmic binding protein *merP*	Mercury	Mercury
		MerA	Mercury (II) reductase	Mercury	Mercury
		MerA	Mercury (II) reductase	Mercury	Mercury
		MerP	Mercury resistance system periplasmic binding protein *merP*	Mercury	Mercury
		MerF	Mercury resistance system transport protein *merF*	Mercury	Mercury
		MerA	Mercury (II) reductase	Mercury	Mercury
		MerP	Mercury resistance system periplasmic binding protein *merP*	Mercury	Mercury
		MerA	Mercury (II) reductase	Mercury	Mercury
		ArsD	Arsenite efflux transporter metallochaperone *arsD*	Arsenic	Arsenite
	D2	*MerR*	Mercury resistance transcriptional regulator *merR*	Mercury	Mercury
		MerT	MerCuric transport protein *merT*	Mercury	Mercury
		MerP	Mercury resistance system periplasmic binding protein *merP*	Mercury	Mercury
		MerC	Organomercurial transporter *merC*	Mercury	Organomercury
		MerA	Mercury (II) reductase	Mercury	Mercury
		MerD	Mercury resistance co-regulator *MerD*	Mercury	Mercury
		MerE	Broad-spectrum Mercury transporter *merE*	Mercury	Mercury

		ArsD	Arsenite efflux transporter metallochaperone *arsD*	Arsenic	Arsenite
		MerE	Broad-spectrum Mercury transporter *merE*	Mercury	Mercury
		MerD	Mercury resistance co-regulator *merD*	Mercury	Mercury
		MerA	Mercury (II) reductase	Mercury	Mercury
		MerF	Mercury resistance system transport protein *merF*	Mercury	Mercury
		MerP	Mercury resistance system periplasmic binding protein *merP*	Mercury	Mercury
		MerT	MerCuric transport protein *merT*	Mercury	Mercury
		MerR	Mercury resistance transcriptional regulator *merR*	Mercury	Mercury
		MerA	Mercury (II) reductase	Mercury	Mercury
		MerP	Mercury resistance system periplasmic binding protein *merP*	Mercury	Mercury
		MerA	Mercury (II) reductase	Mercury	Mercury
		MerP	Mercury resistance system periplasmic binding protein *merP*	Mercury	Mercury
		MerP	Mercury resistance system periplasmic binding protein *merP*	Mercury	Mercury
		MerF	Mercury resistance system transport protein *merF*	Mercury	Mercury
		MerF	Mercury resistance system transport protein *merF*	Mercury	Mercury
		MerA	Mercury (II) reductase	Mercury	Mercury
		MerR	Mercury resistance transcriptional regulator *merR*	Mercury	Mercury
		MerP	Mercury resistance system periplasmic binding protein *merP*	Mercury	Mercury
		MerP	Mercury resistance system periplasmic binding protein *merP*	Mercury	Mercury
		MerF	Mercury resistance system transport protein *merF*	Mercury	Mercury
		MerA	Mercury (II) reductase	Mercury	Mercury
		MerA	Mercury (II) reductase	Mercury	Mercury
		MerA	Mercury (II) reductase	Mercury	Mercury
		MerP	Mercury resistance system periplasmic binding protein *merP*	Mercury	Mercury
		MerF	Mercury resistance system transport protein *merF*	Mercury	Mercury
		ArsD	Arsenite efflux transporter metallochaperone *arsD*	Arsenic	Arsenite
		MerA	Mercury (II) reductase	Mercury	Mercury
		MerA	Mercury (II) reductase	Mercury	Mercury
		MerP	Mercury resistance system periplasmic binding protein *merP*	Mercury	Mercury
		MerA	Mercury (II) reductase	Mercury	Mercury
		MerP	Mercury resistance system periplasmic binding protein *merP*	Mercury	Mercury
		MerA	Mercury (II) reductase	Mercury	Mercury
		MerP	Mercury resistance system periplasmic binding protein *merP*	Mercury	Mercury
		MerD	Mercury resistance co-regulator *merD*	Mercury	Mercury
		MerE	Broad-spectrum Mercury transporter *merE*	Mercury	Mercury
		MerE	Broad-spectrum Mercury transporter *merE*	Mercury	Mercury
		MerD	Mercury resistance co-regulator *merD*	Mercury	Mercury
		MerA	Mercury (II) reductase	Mercury	Mercury
		MerF	Mercury resistance system transport protein *merF*	Mercury	Mercury

		MerP	Mercury resistance system periplasmic binding protein *merP*	Mercury	Mercury
		MerT	MerCuric transport protein *merT*	Mercury	Mercury
		MerR	Mercury resistance transcriptional regulator *merR*	Mercury	Mercury
		ArsD	Arsenite efflux transporter metallochaperone *arsD*	Arsenic	Arsenite
		MerP	Mercury resistance system periplasmic binding protein *merP*	Mercury	Mercury
		MerF	Mercury resistance system transport protein *merF*	Mercury	Mercury
		MerA	Mercury (II) reductase	Mercury	Mercury
		MerP	Mercury resistance system periplasmic binding protein *merP*	Mercury	Mercury
		MerA	Mercury (II) reductase	Mercury	Mercury
		MerA	Mercury (II) reductase	Mercury	Mercury
		MerP	Mercury resistance system periplasmic binding protein *merP*	Mercury	Mercury
		ArsD	Arsenite efflux transporter metallochaperone *arsD*	Arsenic	Arsenite
		MerA	Mercury (II) reductase	Mercury	Mercury
		MerP	Mercury resistance system periplasmic binding protein *merP*	Mercury	Mercury
		ArsD	Arsenite efflux transporter metallochaperone *arsD*	Arsenic	Arsenite
E	E1	*MerP*	Mercury resistance system periplasmic binding protein *merP*	Mercury	Mercury
		MerA	Mercury (II) reductase	Mercury	Mercury
	E2	*MerA*	Mercury (II) reductase	Mercury	Mercury
		ArsD	Arsenite efflux transporter metallochaperone *arsD*	Arsenic	Arsenite
		MerE	Broad-spectrum Mercury transporter *merE*	Mercury	Mercury
		MerA	Mercury (II) reductase	Mercury	Mercury
		MerP	Mercury resistance system periplasmic binding protein *merP*	Mercury	Mercury
		MerA	Mercury (II) reductase	Mercury	Mercury
		MerR	Mercury resistance transcriptional regulator *merR*	Mercury	Mercury
		MerT	Mercuric transport protein *merT*	Mercury	Mercury
		MerP	Mercury resistance system periplasmic binding protein *merP*	Mercury	Mercury
		MerC	Organomercurial transporter *merC*	Mercury	Organomercury
		MerP	Mercury resistance system periplasmic binding protein *merP*	Mercury	Mercury
		MerB	Organomercurial lyase *merB*	Mercury	Organomercury

Reference

ECDC 2019. European Center for Disease Prevention and Control. Antimicrobial consumption in Finland, 2019. https://www.ecdc.europa.eu/en/antimicrobial-consumption/database/country-overview. Adopted in 09.09.2021.
